# Computational assessment of memory function in kidney transplant recipients and donors

**DOI:** 10.1038/s43856-026-01663-x

**Published:** 2026-06-05

**Authors:** Thomas J. Wilschut, Tim J. Knobbe, Andrea Stocco, Holly Hake, Anna M. Posthumus, Aaltje L. Ziengs, TransplantLines Investigators, Tim J. Knobbe, Anna M. Posthumus, Stefan P. Berger, Martin H. de Borst, Rianne M. Douwes, Michele F. Eisenga, Antonio W. Gomes-Neto, Daan Kremer, Marco van Londen, Jan-Stephan F. Sanders, J. Cas Swarte, Caecilia S. E. Doorenbos, Jip Jonker, Charlotte A. te Velde-Keyzer, Isabelle J. C. Dielwart, Coby Annema, Gerard Dijkstra, Rinse K. Weersma, Frank A. J. A. Bodewes, Willem S. Lexmond, Marieke T. de Boer, Vincent E. de Meijer, Arjan Diepstra, Marius C. van den Heuvel, Michiel E. Erasmus, C. Tji Gan, Erik A. M. Verschuuren, Eelko Hak, Frank Klont, Bouke G. Hepkema, Daan J. Touw, Henri G. D. Leuvenink, Hubert G. M. Niesters, Coretta C. van Leer-Buter, L. Joost van Pelt, Michel J. Vos, Robert A. Pol, Adelita V. Ranchor, Marion J. Siebelink, Riemer H. J. A. Slart, Gertrude J. Nieuwenhuis-Moeke, Hans Blokzijl, Kevin Damman, Stephan J. L. Bakker, Hans Blokzijl, Kevin Damman, Vincent E. de Meijer, Martin H. de Borst, Anne M. Buunk, Jacoba M. Spikman, Hedderik van Rijn, Stephan J. L. Bakker

**Affiliations:** 1 Precision Cognition Labs, Groningen, The Netherlands; 2 Department of Experimental Psychology, University of Groningen, Groningen, The Netherlands; 3 Division of Nephrology, Department of Internal Medicine, University Medical Center Groningen, University of Groningen, Groningen, The Netherlands; 4 Department of Psychology, University of Washington, Seattle, WA USA; 5 Department of Neurology, University Medical Center Groningen, University of Groningen, Groningen, The Netherlands; 6 Department of Gastroenterology and Hepatology, University Medical Center Groningen, University of Groningen, Groningen, The Netherlands; 7 Department of Cardiology, University Medical Center Groningen, University of Groningen, Groningen, The Netherlands; 8 Department of Surgery, Section of Hepatobiliary Surgery and Liver Transplantation, University Medical Center Groningen, University of Groningen, Groningen, The Netherlands; 9 Department of Health Sciences, Section of Nursing Science, University Medical Center Groningen, University of Groningen, Groningen, The Netherlands; 10 Division Pediatric Gastroenterology and Hepatology, Department of Pediatrics, University Medical Center Groningen, University of Groningen, Groningen, The Netherlands; 11 Department of Pathology & Medical Biology, University Medical Center Groningen, University of Groningen, Groningen, The Netherlands; 12 Department of Cardiac Surgery, University Medical Center Groningen, University of Groningen, Groningen, The Netherlands; 13 Department of Respiratory Diseases, Tuberculosis and Lung Transplantation, University Medical Center Groningen, University of Groningen, Groningen, The Netherlands; 14 Unit of PharmacoTherapy, -Epidemiology and -Economics, Groningen Research Institute of Pharmacy, University Medical Center Groningen, University of Groningen, Groningen, The Netherlands; 15 Department of Clinical Pharmacy and Pharmacology, University Medical Center Groningen, University of Groningen, Groningen, The Netherlands; 16 Department of Laboratory Medicine, Transplantation Immunology, University Medical Center Groningen, University of Groningen, Groningen, The Netherlands; 17 Department of Pharmaceutical Analysis, Groningen Research Institute of Pharmacy, University of Groningen, Groningen, The Netherlands; 18 Department of Surgery, Surgical Research Laboratory, University Medical Center Groningen, University of Groningen, Groningen, The Netherlands; 19 Department of Medical Microbiology and Infection Prevention, Division of Clinical Virology, University Medical Center Groningen, University of Groningen, Groningen, The Netherlands; 20 Department of Laboratory Medicine, University Medical Center Groningen, University of Groningen, Groningen, The Netherlands; 21 Division of Vascular Surgery, Department of Surgery, University Medical Center Groningen, University of Groningen, Groningen, The Netherlands; 22 Department of Health Psychology, University Medical Center Groningen, University of Groningen, Groningen, The Netherlands; 23 University Medical Center Groningen Transplant Center, University Medical Center Groningen, University of Groningen, Groningen, The Netherlands; 24 Department of Nuclear Medicine and Molecular Imaging, Medical Imaging Centre, University Medical Center Groningen, University of Groningen, Groningen, The Netherlands; 25 Department of Anaesthesiology, University Medical Center Groningen, University of Groningen, Groningen, The Netherlands

**Keywords:** Nephrology, Medical research

## Abstract

**Background:**

Impaired memory function is a frequent yet understudied symptom in kidney transplant recipients. In part, this knowledge gap reflects the lack of scalable, sensitive, and low-burden tools for quantifying memory function in large clinical populations. The aim of this study is to evaluate a brief, remote memory assessment and to examine memory function and its clinical correlates in kidney transplant recipients compared with healthy controls.

**Methods:**

In this cross-sectional study, we demonstrate a remote, minimally burdensome memory screener, the Seattle-Groningen Memory Assessment, which estimates a patient’s speed of forgetting from paired-associate learning. Participants aged 22-86 years from a large transplant cohort completed an eight-minute online memory test. Memory performance was compared between kidney transplant recipients (*n* = 556) and kidney donors (*n* = 408). Associations with demographic, clinical, and physiological variables were examined using regression analyses.

**Results:**

Here, we show that the memory score derived from an eight-minute session is a reliable and accurate measure of an individual’s ability for long-term retention. Kidney transplant recipients show more forgetting than donors. Memory scores are sensitive to demographic factors, including age and education level, and are associated with self-reported sleep quality, fatigue, and health-related quality of life. On the physiological level, more forgetting in recipients is linked to higher monocyte, neutrophil, reticulocyte, and white blood cell counts, as well as lower ferritin and greater iron deficiency.

**Conclusions:**

This work highlights the potential of computational memory assessment as a minimally burdensome and reliable tool for detecting cognitive impairment in complex clinical populations. Such tools may enable scalable monitoring of cognitive health and improve the detection of subtle cognitive changes relevant for disease progression and treatment evaluation.

## Plain Language Summary

Memory problems are commonly reported by people who receive a kidney transplant, but they are often difficult to measure with traditional cognitive or neuropsychological tests. In this study, we evaluated a brief online memory task that can be completed at home in about eight minutes. The test measures how quickly newly learned information is forgotten over time. More than 500 kidney transplant recipients and about 400 kidney donors completed the assessment. We found that transplant recipients, on average, forgot information more quickly than donors. Memory performance was also related to factors such as age, education, sleep quality, fatigue, and several blood markers linked to inflammation and iron levels. Our findings suggest that short digital memory tests may offer a practical way to monitor cognitive health in people with complex medical conditions and could support future research and clinical care.

## Abstract (web summary)

Wilschut, Knobbe et al. evaluate the feasibility of a brief remote memory assessment for large-scale cognitive screening in kidney transplant recipients and donors. They show the method provides reliable measures of forgetting and detects subtle memory differences linked to health and physiological markers.

## Introduction

Cognitive difficulties are a common yet understudied problem in patients with kidney failure and in kidney transplant recipients (KTR)^1,2^. An increasing number of studies report deficits in memory, processing speed, and executive function in patients with kidney failure prior to transplantation as well as in KTR^3–6^. In addition, recent evidence indicates that mild cognitive impairment—a key precursor to Alzheimer’s Disease—affects 16% of outpatient KTR^2^. Research suggests that memory deficits often persist, even after successful kidney transplantation^3,7^. Moreover, studies report significantly elevated risk of neurodegenerative diseases, including Alzheimer’s disease and vascular dementia, in patients with advanced kidney disease^8–10^.

Accurate and accessible screening of cognitive- and memory function is valuable for both clinical practice and for scientific research into the mechanisms that underlie impaired cognitive function in KTR. In practice, however, widely used screening tools like the Mini-Mental State Examination (MMSE)^11^ and Montreal Cognitive Assessment (MoCA)^12^ face some limitations. First, the specificity and sensitivity of these tests to detect abnormalities in memory performance is generally low^13^. Second, their fixed, non-adaptive format may negatively affect the test-taker experience, as it may be overwhelming for patients with cognitive impairments and tedious for individuals without impairments. Third, these tests generally show large practice or test-retest effects that limit repeatability and the ability to track changes in cognitive performance over time^14^.

These methodological shortcomings have tangible consequences for both clinical practice and fundamental research. In clinical practice, quick and accessible screening of cognitive impairment is valuable for assessing the need for further neuropsychological investigation, which in turn could lead to further tailored interventions—such as providing targeted support for medication adherence in patients with memory impairment or offering early cognitive rehabilitation^15^. For fundamental research, the lack of sensitive and repeatable memory assessments limits the ability to track cognitive trajectories over time and, as a consequence, to identify underlying neural or systemic contributors to cognitive decline. In addition, without reliable tools to measure subtle cognitive changes, it is challenging to evaluate the efficacy of targeted interventions.

To address this gap, we use a newly developed digital memory test, the Seattle-Groningen Memory Assessment (SGMA). In this memory test, participants are interleavedly asked to memorize cue-answer pairs and quizzed on their ability to recall the answer when just presented with the cue. The topics can be selected to fit the interest of the participant (and can range from for example animal species to toponymy items). This type of testing resembles flashcard learning (common practice in educational settings). Designed for remote administration and completed in just 8 minutes, SGMA aims to provide reliable and precise assessments of long-term memory function. The assessment is adaptive and tailors the test to be optimally efficient for each participant, making it minimally burdensome. Internally, SGMA relies on computational phenotyping: it uses the patient’s data (accuracy and response times) to fit an established computational model of memory retrieval originally proposed by Anderson and Schooler^16^ and Pavlik and Anderson^17^. The model simulates the accessibility or ‘activation’ of information in the patient’s memory over time. Critically, this approach isolates a central measure of memory function (the speed at which information is forgotten in long-term memory) from other peripheral factors (such as perceptual and motor delays). At the end of the test, a patient’s memory capacity can be summarized in a single idiographic SGMA score, reflecting the average rate at which memory activation declined during the session. The within-session forgetting rates captured by the SGMA score generalize to memory decay over extended periods^18^. As the SGMA score can be calculated based on different item sets and reflects the *process* of memorization instead of the outcome, it avoids practice or test-retest effects and minimizes the influence of participant’s prior knowledge on the test materials [see ref. ^19^].

In earlier work, the SGMA score has been shown to outperform other cognitive screening outcomes, such as General Cognitive Capacity (GCA, a measure similar to a general intelligence or IQ score) and Working Memory Capacity (WMC), in predicting long-term memory recall^20^. Neuroimaging studies indicate that the SGMA score is linked to distributed resting-state activity in EEG and fMRI data^21,22^, which is known to capture stable individual differences in brain and cognitive function. Recent clinical research has demonstrated robust correlations between the SGMA score and existing assessments memory and cognitive function, including the Consortium to Establish a Registry for Alzheimer’s Disease (CERAD) and Montreal Cognitive Assessment (MoCA)^23^. A large-scale study in collaboration with Seattle’s Alzheimer’s Disease Research Center (ADRC) and the University of Washington found that the SGMA score derived from a single 8-minute session completed at home achieved 82% accuracy in detecting mild cognitive impairment^19^. Overall, early findings suggest that the SGMA score is a fast and reliable measure of a trait-like cognitive capacity that uniquely predicts an individual’s ability for long-term retention.

In this study, we employ the SGMA to investigate memory function in KTR by conducting a cross-sectional, online behavioral study involving 408 KTR and 556 (potential) kidney donors (KD) from the TransplantLines Biobank and Cohort Study [refs. ^24, 25^, see Supplementary Fig. 1]. The study has two primary aims. First, we evaluate the clinical feasibility, validity, and reliability of SGMA for large-scale memory screening. To do this, we analyzed patient response rates, SGMA’s sensitivity to demographic variables, its correlations with established neuropsychological assessments, and its associations with subjective memory ratings. We additionally included self-report measures of sleep, fatigue, and general health to capture everyday factors that may influence cognitive functioning. Second, we explore how various pre- and post-transplant health factors relate to memory function in KTR, using the SGMA scores in combination with clinical, demographic, and biochemical data, and compared these patterns with those observed in (potential) KD. While directly relevant to the KTR population, this work will also provide insights to support the development of improved cognitive assessments for use in other complex, multimorbid populations.

To foreshadow the results, in this study, we show that the SGMA provides a reliable and scalable measure of long-term memory function in a large clinical cohort. Kidney transplant recipients exhibit faster forgetting compared with (potential) kidney donors. Memory scores vary systematically with demographic characteristics such as age and education, and are associated with self-reported sleep quality, fatigue, and health-related quality of life. In addition, memory performance relates to several physiological markers, including inflammatory cell counts and indicators of iron status. Together, these findings demonstrate the feasibility of brief computational memory assessments for large-scale screening and highlight their potential to detect subtle cognitive differences relevant to clinical populations such as kidney transplant recipients.

## Methods

### Participants

An invitation to complete the three SGMA sessions was sent to all enrolled and alive participants of the TransplantLines Biobank and Cohort study^24^. This study, initiated in June 2015, invited all (potential) solid organ transplant recipients and donors (≥ 18 years old) from the University Medical Center Groningen (UMCG) to participate (participation rate of 80%). Written informed consent was obtained before inclusion. The TransplantLines study protocol was approved by the University Medical Center Groningen Institutional Review Board (METc 2014/077), adheres to the UMCG Biobank Regulation, and is in accordance with the WMA Declaration of Helsinki and the Declaration of Istanbul^24^. The study protocol has been described previously in Eisenga et al.^24^ and Posthumus et al.^25^.

For the current study, we included all KTR or (potential) KD who completed at least one complete SGMA session that fell outside of the exclusion criteria (see section “Seattle-Groningen Memory Assessment”). This resulted in a sample of 556 KTR and 408 (potential) KD (see section “Baseline characteristics”).

### Procedure

Eligible participants of the TransplantLines Biobank and Cohort Study (see section “Participants”) were invited using the REDCap survey and database managing software^26^. In total, participants were invited to up to three measurement sessions via email. The first invitations to participate were sent on July 17th, 2024. Before starting the first memory measurement (see section “Seattle-Groningen Memory Assessment”), participants were asked to complete three questionnaires on general well-being (see section “ Patient-reported outcome measures”). Exactly 7 days after completion of the first memory test, participants received an invitation to participate in the second memory test. Likewise, exactly 7 days after completion of the second memory test, an invitation for the third test followed. Participants who did not complete the test received a first reminder three days after each invitation, and a second reminder three days after that. A timeline of the most important events in this study is summarized in Supplementary Fig. 2.

### Seattle-Groningen memory assessment

Participants received invitations to complete three digital SGMA sessions, spaced one week apart. Participants were able to choose from an array of test topics based on personal preference, including Architecture, Dog breeds, Cheeses, Castles, Mushrooms, Pasta, Constellations, Flags, and Sailing Knots (participants could not choose the same topic for multiple tests). SGMA sessions took 8 minutes to complete, and within each session, items were adaptively introduced based on the performance of the participants. For each item, response latencies and accuracy scores were recorded and used to estimate a *speed of forgetting* parameter, which was continuously updated throughout the session. The final speed of forgetting parameter estimated at the end of the session, averaged over all items, was converted to a SGMA score using a sigmoid function (for more details on the adaptive item scheduling algorithm and the estimation of the speed of forgetting from responses, see Hake, Velde, Leonard, Grabowski, Rijn, and Stocco^27^ and Sense, Behrens, Meijer, and Rijn^19^. To improve reliability and reduce measurement error, SGMA scores were calculated as the average across all completed sessions (up to three per participant), thereby reducing the influence of transient factors and random variability on the estimated memory outcome.

SGMA sessions were screened for irregularities to ensure high data quality, particularly given the online nature of data collection. We applied relatively strict exclusion criteria for this purpose. Sessions that included a pause exceeding 60 seconds were excluded, as long interruptions or distractions during the task compromise the accurate estimation of forgetting speed over time. Similarly, sessions that were terminated after less than 6 minutes were removed, as they reflect incomplete data or disengagement. We also excluded sessions with an average accuracy below 50.00%, and sessions with a median response time above 8 seconds, which may indicate non-compliance, a lack of understanding of the task, or extreme inattentiveness.In total, 11.80% of all trials were excluded based on these above criteria. The applied performance criteria (for average accuracy and median response times) were intentionally designed to be very lenient in order to only exclude sessions reflecting disengagement, a misunderstanding of the task, or interruptions during testing rather than poor memory performance per se. From 12 KTR and 14 (potential) KD, data for at least one memory assessment was removed based on the performance criteria. From 6 KTR and 1 KD, data from two sessions were removed.

To further ensure comparability across individuals and sessions, all SGMA scores were adjusted for average topic difficulty, using the residuals of a linear regression model estimating SGMA score from test topic.

### Neuropsychological tests

Between June 2015 and March 2021, participants of TransplantLines were randomized to undergo either an extensive physical or neuropsychological assessment. Consequently, a subset of the participants who completed the SGMA had completed a series of neuropsychological tests during this period. These tests were administered by a trained researcher with a background in psychology and typically took between 30 and 45 min to complete. The tests were conducted a median of 6.26 years (*IQR* = 5.77−7.22 years) prior to completion of the first memory test. The following constructs were included in the assessment:

*Attention* The Trail Making Test part B (**Trail Making Test-B**) assesses attention, cognitive flexibility, executive functioning, and task-switching ability. Participants are asked to connect alternating sequences between numbers and letters in ascending order (e.g., 1-A-2-B-3-C…). The task requires maintaining attention while switching between sequences. The completion time is recorded, with faster times indicating better performance^28^.

*Executive functioning* The **Clock Drawing** Test assesses visuospatial skills, executive function, and memory. Participants draw a clock, place numbers correctly, and set the hands to a given time. Errors in layout, sequencing, or time placement can indicate cognitive impairment. Scoring is based on accuracy, with points assigned for correct shape, number placement, and hand positioning^29^.

*Memory* The Wechsler Adult Intelligence Scale IV is an intelligence test for adults that includes working memory tests. One of these sub-tests, the Digit Span, has two parts: in the first part, the participant must repeat a sequence of digits in the same order, which tests working memory (**Digit Span Forwards**). In the second part, they repeat the digits in reverse order, resulting in a more demanding test of working memory (**Digit Span Backwards**)^30^.

The 15 Words Test (**15 Words Test**) evaluates verbal memory. Participants are presented with a list of 15 unrelated words over five consecutive trials and must recall as many as possible immediately after each trial (Immediate recall). A delayed recall (Delayed retention) test is also conducted after 20 min^31^.

Language **Word fluency** is a verbal task assessing language fluency and semantic memory. Participants must generate as many words as possible within a given category (words with a specific first letter (l), certain professions (p) and animals (a)) in one minute, and their total score is based on the number of words produced^32^.

The **Dutch Reading Test** for Adults is a test used to estimate the general intelligence and cognitive function. The test consists of a series of words with irregular pronunciation, meaning they do not follow the usual pronunciation rules. The patient is required to read these words aloud. The NLV is a validated estimate of (verbal) intelligence. The score is determined by the number of correctly pronounced words^33^.

Processing speed Trail Making Test part A (**Trail Making Test-A**) measures mental speed and involves connecting 25 numbers in ascending order, as quickly as possible. Completion of the tests is timed in number of seconds^28^.

The **Symbol Digit Modalities Test** assesses processing speed, attention, and cognitive flexibility. Participants match symbols to numbers based on a provided key, within a time limit. The score is based on the number of correct matches, with faster and more accurate responses indicating better cognitive function^34^.

### Patient-reported outcome measures

Fatigue was measured using the validated 8-item Checklist Individual Strength Revised (CIS8R), which evaluates subjective fatigue based on eight questions regarding fatigue intensity over the past two weeks^35,36^. A higher score indicates greater fatigue. Sleep quality was assessed using the 19-item Pittsburgh Sleep Quality Index (PSQI), which measures sleep quality and disturbances across seven domains^37,38^. A higher score indicated poorer sleep quality. Health-related quality of life (HRQoL) was assessed using the commonly applied Short Form 12 (SF12), a reliable and validated 12-item instrument, resulting in 8 subscales, which can be combined into a physical and mental component score^39–41^. A higher score indicates better HRQoL.

### Demographic, clinical, and laboratory variables

Demographic and transplant-related data were collected by questionnaires or extracted from the patient’s medical records. Sex was defined as sex assigned at birth. For commonly performed laboratory measurements in clinical care (i.e., leukocytes, estimated glomerular filtration rate, albumin, hemoglobin, sodium, creatinine, glucose, urea) the most recent result was extracted from the patient’s medical records. For the other laboratory measurements, which are less frequently assessed in clinical care, results from the most recent TransplantLines study visit were used to minimize indication bias. For recipients, median time between data extraction from the medical file and the SGMA was 0.12 years (*IQR* = 0.03−0.44); for lab results from the latest study visit, 2.73 years (*IQR* = 0.74−6.09). For donors, the median time between medical file data and the SGMA was 0.74 years (*IQR* = 0.36−1.78); for study visit lab results, 2.65 years (*IQR* = 1.22−4.08). Laboratory measurements were performed using routine laboratory methods. eGFR was calculated using the Chronic Kidney Disease Epidemiology Collaboration equation^42^. Iron deficiency was defined using both ferritin and transferrin saturation levels. For kidney donors, iron deficiency was defined as ferritin levels below 30 μg/*L* in males and below 15 μg/*L* in females. For recipients, iron deficiency was defined by a combination of ferritin levels below 100 μg/*L* and transferrin saturation below 20% ^43,44^. Individuals not meeting these criteria were classified as not iron deficient. Medication use was extracted from the patient’s medical records.

### Statistics and reproducibility

For all sections, data preprocessing and statistical analyses were conducted with R [v.4.3.1,^45^]. All data was visualized using ggplot2 [v.3.5.1,^46^]. Regression analyses were conducted using *lme4* [v.1.1-34,^47^] and *lmerTest* [v.3.1-3^48^] packages.

Comorbidities were not included as covariates in the primary analyses. The primary aim of the study was to compare cognitive performance between KTR and (potential) KD at the population level, thereby capturing the combined cognitive impact of kidney failure, transplantation-related factors (e.g., immunosuppressive treatment), and co-occurring health conditions that are intrinsic to the clinical phenotype of KTR. Adjusting for comorbidities would therefore have removed part of the clinically relevant variance of interest.

To generate the results reported in section “Physiological markers”, we calculated univariable pearson’s correlations between (a) the SGMA score for recipients, (b) the SGMA score for recipients adjusted for demographics age, sex and education level, (c) the SGMA score for donors, and (d) the SGMA score adjusted for demographics age, sex, and education level using available blood markers in the TransplantLines Biobank. If necessary (skewness  > 2 or  < − 2) lab markers were converted to normal distributions using a log-transform. All lab variables were standardized and adjusted for sex. Lab measurements were included if they contained less than 20% missing data in the sample of participants. The remaining number of observations per blood marker is shown in Supplementary Table 5. No formal correction for multiple testing (e.g., Bonferroni or FDR) was applied to the univariable correlation analyses with physiological markers, as these were considered exploratory and intended to identify potential associations rather than a set of predefined hypotheses.

To mitigate the risk of overfitting and spurious findings, these analyses were complemented with LASSO regression models, which perform variable selection and shrinkage to provide more conservative estimates of the contribution of physiological markers to memory performance. We fitted four variations of penalized regression (LASSO) models using R package *glmnet* [v.4.1-8,^49^] to predict outcome measures (a), (b), (c) and (d) mentioned above. The LASSO model was fitted using 100-fold cross-validation to choose the most optimal elastic net hyper-parameters for mixture (alpha) and penalty (lambda). We report standardized LASSO regression coefficients and R-squared values for all four models.

## Results

### Baseline characteristics

#### Response- and return rates suggest high engagement

The first aim of this project was to assess the feasibility of using SGMA as an online memory screener. All 2301 KTR, KD, or individuals that were undergoing screening for donation in the TransplantLines Biobank and Cohort study were invited by e-mail to complete up to three repeated, online memory assessments. Out of the invited participants, 964 completed at least one SGMA session, corresponding to a 41.89% overall response rate of (see Supplementary Fig. 1). Of all participants who completed the first assessment, 77.05% completed the second assessment and 70.43% completed the third assessment, showing high engagement, low attrition rate, and willingness to return for repeated testing.

#### Comparison of responders and non-responders

Responders are those who completed at least one full memory assessment, whereas non-responders are those who did not. Sample characteristics for responders versus non-responders, for both recipients and (potential) donors, are listed in Supplementary Table 1. In the KTR group, we found no significant differences in terms of age (*p* = 0.773), sex (*p* = 0.179), years since transplantation (*p* = 0.941), pre-transplant dialysis or no pre-transplant dialysis (*p* = 0.081) or kidney function (estimated glomerular filtration rate (eGFR), *p* = 0.496) between responders and non-responders. Responders had more often received a kidney from a living (rather than deceased) donor than non-responders did (61.87% versus 54.99%, *p* = 0.009). In the (potential) KD group, responders were slightly older than non-responders (63.27 versus 60.58 years, *p* < 0.001), and had a lower kidney function (eGFR, 63.09 ± 13.80 versus 68.57 ± 16.92 mL/min/1.73 m^2^, *p* < 0.001). We found no significant differences in sex and time since donation for those who donated. Overall, the comparison between responders and non-responders suggests that the sample was fairly representative of all KTR and KD in the TransplantLines Biobank and Cohort Study.

#### Comparison of recipients and (potential) donors

To assess the memory function of KTR, we compared them to a control group. The control group comprised both individuals who had already donated a kidney and individuals still undergoing screening for donation at the time of assessment. We included both groups in the control sample, as both groups are typically comparable to recipients in geographic background, socio-economic context and age, and represent a relatively healthy population without kidney failure, dialysis exposure, or exposure to immunosuppressive therapy. (Accordingly, individuals in screening for donation and individuals who actually donated did not differ in SGMA scores (*t* = − 0.37, *p* = 0.713.)

Table 1 shows the baseline characteristics for KTR and KD separately. In total, the sample consisted of 556 KTR and 408 (potential) KD. The latter group comprised 357 individuals who donated at the time of participation, and 51 individuals who were being screened for donation. On average, recipients were slightly younger (58.59 ± 12.64 versus 63.27 ± 10.77 years, respectively, *p* < 0.001) and more frequently male (60.01% versus 44.12% respectively, *p* < 0.001) than (potential) KD. Median time since donation or transplantation was higher in recipients than in donors (6.06, *IQR* = 2.50−11.32 versus 4.97, *IQR* = 2.22−7.72 years, respectively, *p* < 0.001). For recipients, the mean eGFR was 53.16 ± 18 mL/min/1.73 m^2^, 62.05% of KTR received a kidney from a living donor and 55.94% underwent dialysis before transplantation.

**Table 1:** Sample characteristics for recipients and (potential) donors

	Recipient	(Potential) donors	*p*
*N*	556	408	
Age (years)	58.59 (12.64)	63.27 (10.77)	<0.001
N female	222 (40%)	228 (56%)	<0.001
Education			
No education	4 (1%)	0 (0%)	
Lower education	13 (2%)	4 (1%)	
Lower vocational education	89 (16%)	47 (12%)	
Intermediate secondary education	157 (28%)	110 (27%)	
Intermediate vocational education	67 (12%)	61 (15%)	
Higher secondary education	44 (8%)	32 (8%)	
Higher vocational education	136 (24%)	120 (29%)	
Academic education	46 (8%)	34 (8%)	
Years since donation or transplantation	6.06 (2.50–11.32)	4.97 (2.22–7.72)	<0.001
N living donor	345 (62%)		
N pre-transplant dialysis	311 (56%)		
eGFR^a^ (mL/min/1.73 m^2^)	53.16 (17.53)	63.09 (13.80)	<0.001

Time since donation or transplantation shows median (IQR) values, the other rows show means (SD) or absolute numbers and proportions. Differences between donors and recipients were assessed using two-sided Welch two-sample t-tests. Bold p-values indicate statistical significance (*p* < 0.05).

^a^estimated glomerular filtration rate (creatinine).

### Validity and reliability

#### SGMA scores are sensitive to demographic variables

**Fig. 1:**
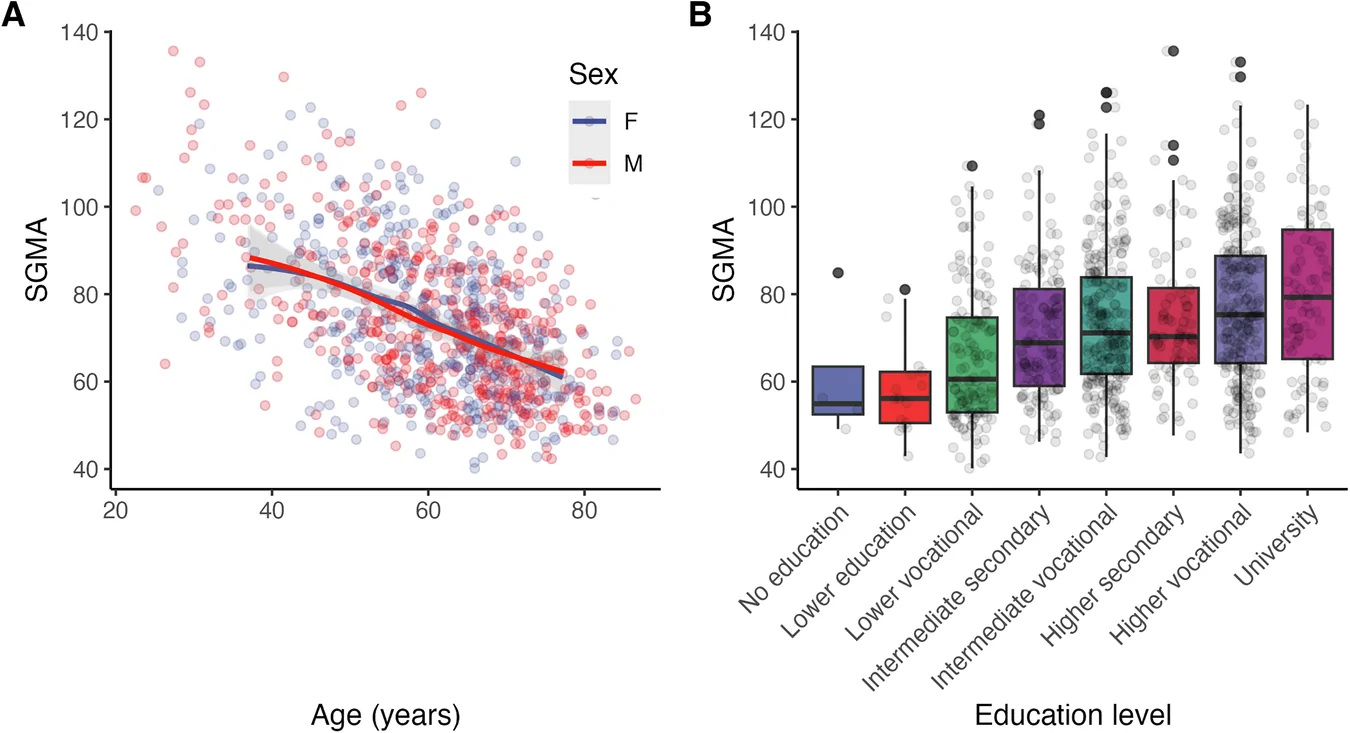
Memory performance in KTR and (potential) KD, based on SGMA scores adjusted for age, sex, and education (SGMAa). **A** KTR show significantly lower SGMAa scores compared to (potential) KD, indicating reduced memory performance in the recipient group. Box plots show the median (horizontal line), interquartile range (box), range (whiskers, extending to 1.5 × IQR), and individual outliers (points beyond whiskers). *n* = 556 biologically independent kidney transplant recipients and n = 408 biologically independent (potential or previous) kidney donors. **p  < 0.01, from a two-sided Welch’s t-test. **B** The SGMAa scores were not significantly associated with time since transplantation or donation. Lines represent linear regression fits; the shaded area indicates the 95% confidence interval around the fitted line.

To assess the validity of the SGMA, we first examined its associations with demographic variables. Figure 1 shows SGMA scores by age, sex, and education level. Panel A demonstrates that SGMA scores, predictably, declined substantially with age. There was no noticeable association with sex. Panel B shows that higher education levels were generally associated with better performance on the memory test. The above results were corroborated by the results of a linear regression model (Table 2), which showed a significant negative association between age and SGMA score (*β* = −0.69, *p* < 0.001), a significant positive association with education level (*β* = 2.34, *p* < 0.001), and no association with sex (*β* = − 0.27, *p* = 0.765). These results align with previous studies linking age^50,51^ and education^52^ to memory performance. Although prior research has occasionally reported sex differences in memory—for example, women are sometimes reported to outperform men on verbal memory tasks, while men may show advantages in spatial memory^53,54^—these effects are typically small. Moreover, because the SGMA is designed to assess memory for image-term associations that are neither purely verbal nor spatial, it is not surprising that no robust sex differences in memory performance were observed in this study.

**Table 2:** Linear regression model for the association of age, sex, and education with the SGMA score

	Estimate	Std. Error	*t*	*p*
(Intercept)	102.50	2.80	36.55	**<0.001**
Age	−0.69	0.04	−18.45	**<0.001**
Sex (female = 0, male = 1)	−0.27	0.91	-0.30	0.76
Education level	2.34	0.27	8.81	**<0.001**

Bold p-values indicate statistical significance (*p* < 0.05).

#### SGMA scores are reliable and show no test-retest effects

**Fig. 2:**
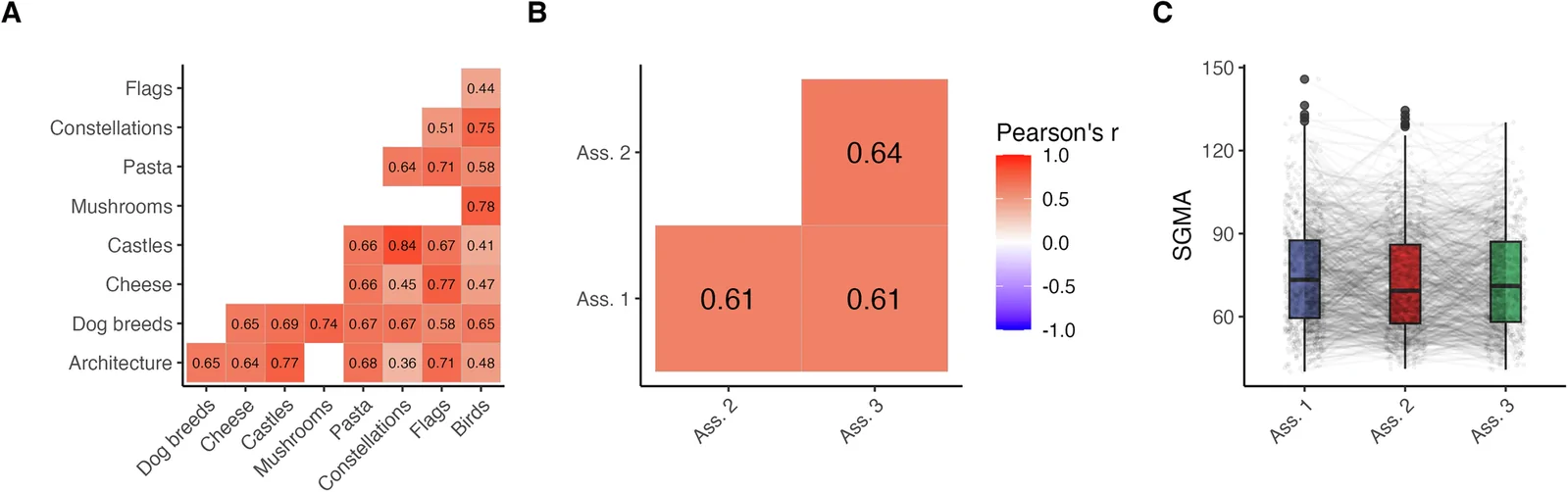
Test-retest reliability and score stability of SGMA assessments across topics and time. **A** shows average test-retest reliability for diff erent test topics for cells for which there were 20 or more pairwise observations. **B** shows average test-retest reliability for assessments. **C** shows the SGMA score distribution over assessments, reflecting the absence of practice or test-retest effects. Lines connect observations of individual participants. *n* = 964 biologically independent participants contributing to repeated SGMA assessments (Assessment 1: n = 906; Assessment 2: *n* = 699; Assessment 3: *n* = 660).

For each of the three memory assessments, participants could choose their own test topic from a list of ten options (see Fig. 2A) based on their personal interest. Figure 2A shows the average correlation between scores on the different topics, and panel B shows the average correlation between the SGMA scores for the three assessments. The reliability of the repeated measurements ranged between 0.61 and 0.64 over sessions, which is typically interpreted as good reliability^55^. Panel C shows the average estimated SGMA scores over assessments, showing that participants did not meaningfully or significantly improve with repeated testing (in fact, average performance showed a nonsignificant *decreasing* trend over assessments, with 0.91 SGMA points per session, *p* = 0.062). Overall, these results confirm that the SGMA score is a stable and repeatable measure of memory performance.

#### SGMA scores are related to memory, processing speed, and attention

**Fig. 3:**
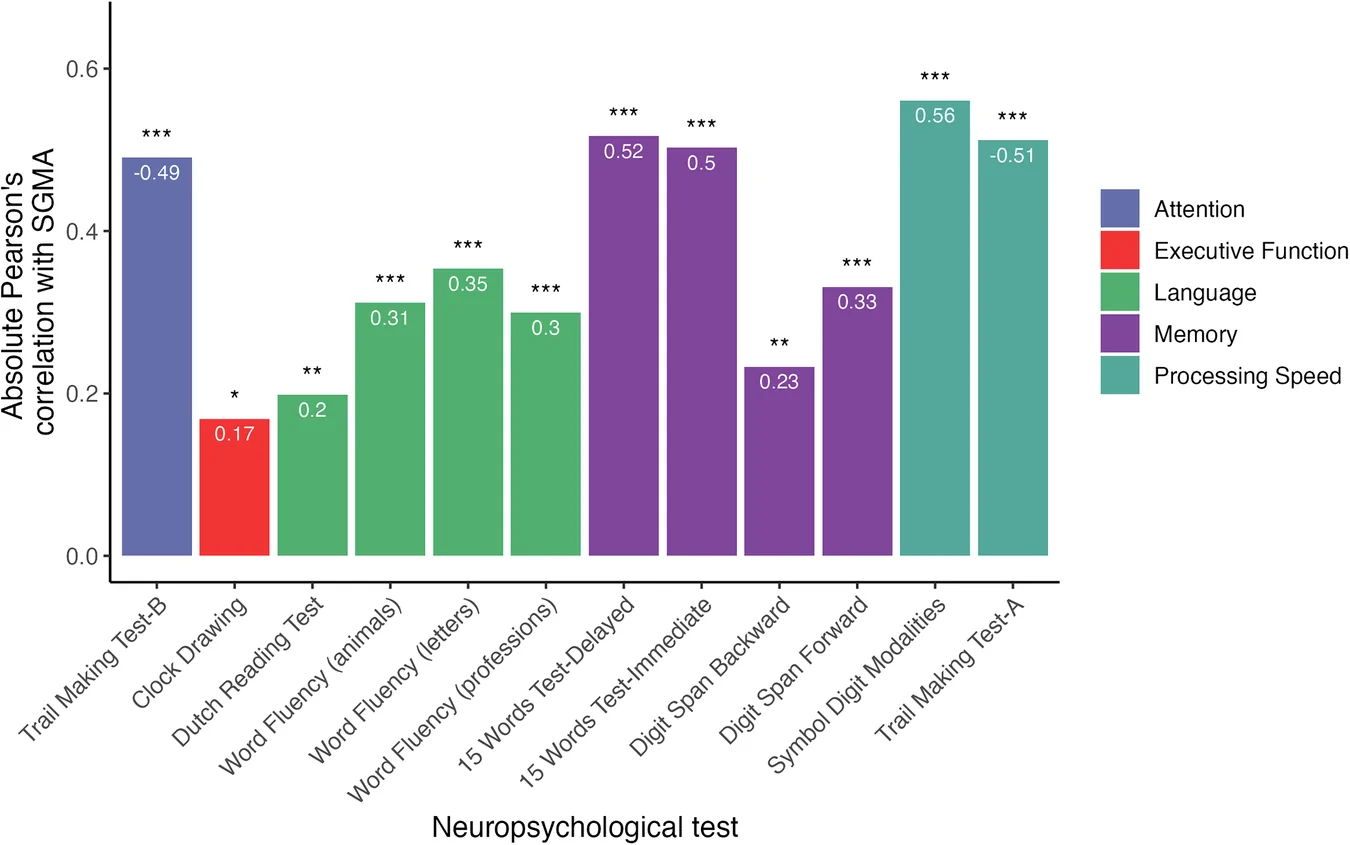
Correlations between neuropsychological tests and the SGMA score. ***: *p* < 0.001; **: *p* < 0.01; *: *p* < 0.05. *n* = 179 biologically independent participants.

To determine the construct validity of the SGMA in the current clinical sample, we analyzed its associations with a battery of established neuropsychological assessments. In total, neuropsychological assessment data was available for 135 KTR and 44 (potential) KD. Figure 3 and Table 3 show Pearson’s correlations between scores on the neuropsychological tests and the SGMA scores. Mean outcome values for neuropsychological tests for recipients and donors separately are provided in Supplementary Table 2.

**Table 3:** Pearson’s correlations of previously performed neuropsychological tests with the SGMA score

Category	Test	Correlation with SGMA score	*p*
Attention	Trail Making Test-B	−0.49*	**<0.001**
Executive Function	Clock Drawing	0.17	**0.030**
Language	Dutch Reading Test	0.20	**0.010**
Language	Word fluency (animals)	0.30	**<0.001**
Language	Word fluency (letters)	0.35	**<0.001**
Language	Word fluency (professions)	0.31	**<0.001**
Memory	15 Words Test - Delayed retention	0.52	**<0.001**
Memory	15 Words Test - Immediate recall	0.50	**<0.001**
Memory	Digit Span Backwards	0.23	**<0.001**
Memory	Digit Span Forwards	0.33	**<0.001**
Processing Speed	Symbol Digit Modalities	0.56	**<0.001**
Processing Speed	Trail Making Test-A	−0.51^a^	**<0.001**

^a^Negative correlations with the SGMA score and SGMAa score align with expectations, as scores on the Trail Making Test (A/B) represent the time needed to complete the task (i.e., longer times indicate worse performance). Correlations were not corrected for multiple comparisons.

Bold p-values indicate statistical significance (*p* < 0.05).

In line with our theoretical framework, SGMA scores showed moderate correlations with key measures of declarative memory, including both immediate and delayed recall on the 15 Words Test (where participants are presented with a list of 15 words, and must recall as many as possible immediately after each trial, and after a delay of 20 minutes). Next to associations with primary measures of declarative memory we observe moderate associations with attention (Symbol Digits Modalities Test, Trail Making Test-A), and processing speed (Trail Making Test-B): constructs that are theoretically and empirically linked to memory storage and retrieval^56,57^. Correlations with language (Word Fluency, Reading) and executive functioning (Clock Drawing) tasks are notably weaker. This difference suggests that the SGMA score does not reflect general cognitive ability, but instead captures key mechanisms related to memory processes.

It is good to note that neuropsychological tests were completed several years prior to the memory assessment during a study visit for the TransplantLines Biobank and Cohort study (median time since completio*n* = 6.26 years, *IQR* = 5.77−7.22 years). As it is possible that at least some of these neuropsychological measures changed in a nonlinear manner during the years between assessments, the absolute strength of the observed correlations may need to be interpreted as conservative *under*estimations of the true associations. However, Supplementary Fig. shows that the strength and direction of correlations between SGMA and neuropsychological test scores were comparable across subgroups split by time since neuropsychological assessment, indicating minimal influence of assessment recency on the observed associations. This suggests that the associations with SGMA scores are robust, supporting the interpretation that the SGMA captures stable, trait-like differences in memory function.

#### The accuracy of memory self-assessment declines with age

Before starting the first memory assessment, all participants were asked to provide a subjective assessment of their own every-day memory performance. Responses were provided (on a 4-point Likert scale) with options (1) ‘poor’; (2) ‘average’, (3) ‘good’; (4) ‘very good’. Table 4 shows the results of a linear regression model estimating the observed SGMA score from subjective memory rating (1–4) and age (also see Supplementary Fig. 4). We found a significant association between subjective memory and the SGMA score: people are able, at least to some extent, to rate their own memory function (*β* = 9.94, *p* < 0.001). There is also an association with age, indicating that older people rate their memory function lower (*β* = − 0.42, *p* < 0.001). Finally, we find an interaction between age and subjective memory, indicating that the association between age and the SGMA score is weaker for people with lower subjective memory ratings (*β* = − 0.13, *p* < 0.006). In other words, older people made less accurate judgments about their own memory function.

**Table 4:** Linear regression model, estimating the SGMA score using subjective memory rating and age

	Estimate	Std. Error	*t*	*p*
(Intercept)	92.51	7.74	11.94	**<0.001**
Subjective memory rating	9.94	2.84	3.50	**<0.001**
Age	−0.42	0.12	−3.37	**0.001**
Subjective memory rating × Age	−0.13	0.05	−2.78	**0.006**

Bold p-values indicate statistical significance (*p* < 0.05).

### Interim summary

Together, the results from the preceding sections demonstrate that SGMA is a feasible, reliable, and valid tool for assessing memory function in KTR and KD. Using SGMA resulted in high response and return rates, suggesting that it is well-received and accessible in clinical populations. The SGMA score was reliable across repeated sessions and showed no practice effects. SGMA scores were sensitive to known demographic predictors of memory performance, and showed robust correlations with established neuropsychological measures—particularly those indexing verbal memory, processing speed, and attention. Overall, by establishing the SGMA as a reliable and valid tool for remote memory assessment, these results provide a foundation to examine how clinical and physiological factors contribute to memory function in KTR-the second aim of this study.

### Associations with clinical variables

#### KTR show lower memory performance than (potential) KD

**Fig. 4:**
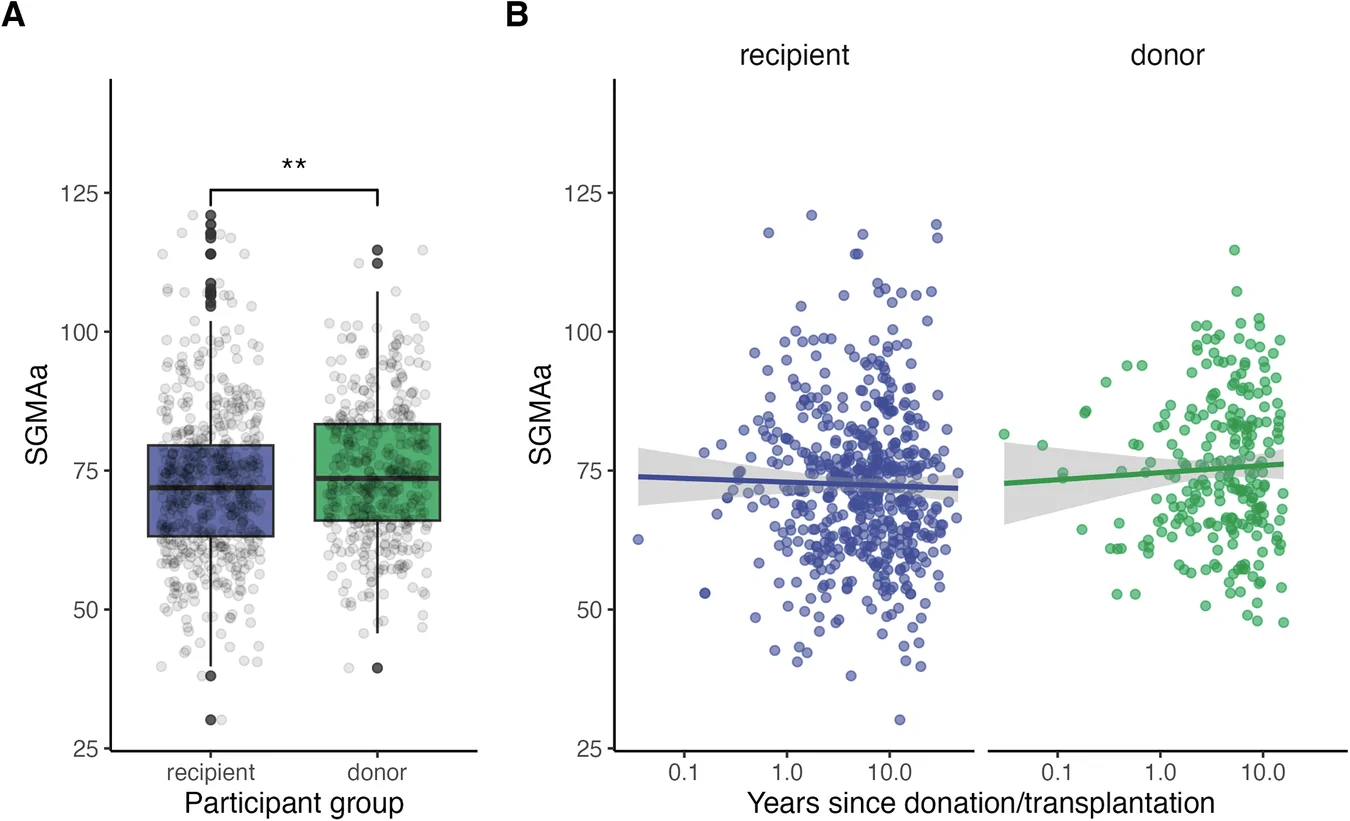
Memory performance in KTR and (potential) KD, based on SGMA scores adjusted for age, sex, and education (SGMAa). **A** KTR show significantly lower SGMAa scores compared to (potential) KD, indicating reduced memory performance in the recipient group. n = 556 biologically independent kidney transplant recipients and n = 408 biologically independent (potential or previous) kidney donors. **p < 0.01, from a two-sided Welch’s t-test. **B** the SGMAa scores were not significantly associated with time since transplantation or donation. The shaded area indicates the 95% confidence interval around the estimated curve.

To properly compare KTR and KD while accounting for demographic confounds, we created an adjusted version of the SGMA score, referred to as SGMA-adjusted (SGMAa), in which the SGMA score is adjusted for age, education and sex. This adjusted score was calculated using the residuals of the linear model shown in Table 2. We compared the memory performance of KTR with (potential) KD using the SGMAa score (thus adjusted for demographic variables); Fig. 4 provides an overview of the results. Panel A shows that recipients had significantly lower SGMAa scores compared to (potential) donors (mean difference = 2.19, *p* = 0.010). This result can be interpreted as a small but statistically significant reduction in memory performance compared to healthy individuals, potentially reflecting the lasting cognitive impact of kidney failure, comorbidities, or factors related to the transplantation (e.g., immunosuppressive treatment). Panel B shows that SGMAa scores were not significantly associated with time since transplantation or donation (recipients: *p* = 0.423, donors: *p* = 0.816). In other words, these results suggest that neither kidney transplantation nor donation influences the normal age-based decline in memory performance. This suggests that the observed lower memory scores in KTR are unlikely to reflect progressive post-transplant cognitive decline, but rather reflect the impact of pre-transplant health factors (note that Fig. 4B shows the age-adjusted SGMA scores, and therefore no decline over time as a result of normal aging is shown. The flat lines in the figure show that there is no *steeper* decline in memory function post transplantation).

To further validate these findings, we conducted a sensitivity analysis comparing KTR with their living KD, in order to achieve better demographic comparability in terms of age, time since transplantation/donation, and geographical area (e.g., often sharing the same household). An independent-samples t-test which included 96 KTR and 96 KD, yielded results consistent with the primary analysis, with significantly lower SGMAa scores among KTR compared to their living kidney donors (*p* = 0.028), as shown in Supplementary Fig. 5. This reinforces the conclusion that the observed differences in memory function are not explained by demographic confounding.

Recipients with Cystic kidney disease as etiology of their kidney failure had higher SGMAa scores than recipients with diabetic nephropathy as etiology of their kidney failure (*p* = 0.028). No differences were between recipients with other etiologies of their kidney failure were found, as presented in Supplementary Table 3 and Supplementary Fig. 6. Within the recipient group, we found no significant difference in the SGMAa scores between those who had received pre-transplant dialysis and those who had not (*p* = 0.867). Similarly, no significant difference was observed between recipients of a kidney from a living versus a deceased donor (*p* = 0.173). Our findings point to a broader cognitive vulnerability that persists post-transplantation. However, we note that the time since transplantation was variable across individuals, and subtle progressive effects or delayed recovery processes cannot be ruled out based on the current results.

#### Immunosuppressive medication in recipients

To examine whether immunosuppressive medication use was related to memory performance. No significant differences in adjusted SGMA scores were found for calcineurin inhibitors (*p* = 0.72), mTOR inhibitor (*p* = 0.07), proliferation inhibitor (*p* = 0.76), or corticosteroid (*p* = 0.92) use. These null findings likely reflect the limited variability in medication regimens—most recipients used similar combinations of immunosuppressants-which reduces the likelihood of detecting differential cognitive effects across groups.

#### Self-reported sleep quality, fatigue, and HRQoL are associated with SGMAa scores

To explore how general well-being relates to memory performance, we examined associations of self-reported measures of sleep quality, fatigue, and health-related quality of life (HRQoL), collected at the time of the third memory assessment, with SGMAa scores. In total, 395 KTR and 314 (potential) KD completed the self-report measures for sleep quality, fatigue, and health-related quality of life. Sleep quality was measured using the Pittsburgh Sleep Quality Index (PSQI), where higher scores indicate poorer sleep. We observed a modest but significant negative correlation with SGMAa (*r* = −0.13, *p* = 0.003), suggesting that individuals who reported poorer sleep tended to show lower memory performance. Fatigue was assessed with the Checklist Individual Strength Revised (CIS-8), where higher scores reflect greater fatigue. A similar pattern emerged, with higher fatigue levels associated with lower SGMAa scores (*r* = − 0.09, *p* = 0.017). Finally, we measured HRQoL using the Short Form 12 (SF-12). Here, we found a positive correlation with the SGMAa for the physical component score (*r* = 0.17, *p* < 0.001), indicating that individuals who perceived their overall physical health and functioning more positively performed better on the memory test. We also found a trend in the direction of an association for the mental component score, with higher scores related to better memory performance (*r* = 0.07, *p* = 0.054). Although the above-mentioned associations are relatively small, they are consistent for reported sleep quality, fatigue, and HRQoL. Together, they suggest that broader aspects of health and daily functioning are related with memory function as captured by the SGMA, also when partialling out potential effects of demographic factors.

### Physiological markers

We analyzed the associations between commonly used biomarkers, collected either during routine clinical care or at the last TransplantLines study visit (see section “Participants”), and memory function to gain insight into long-term physiological processes that may affect memory function.

#### Blood markers explain variance in memory scores

**Fig. 5:**
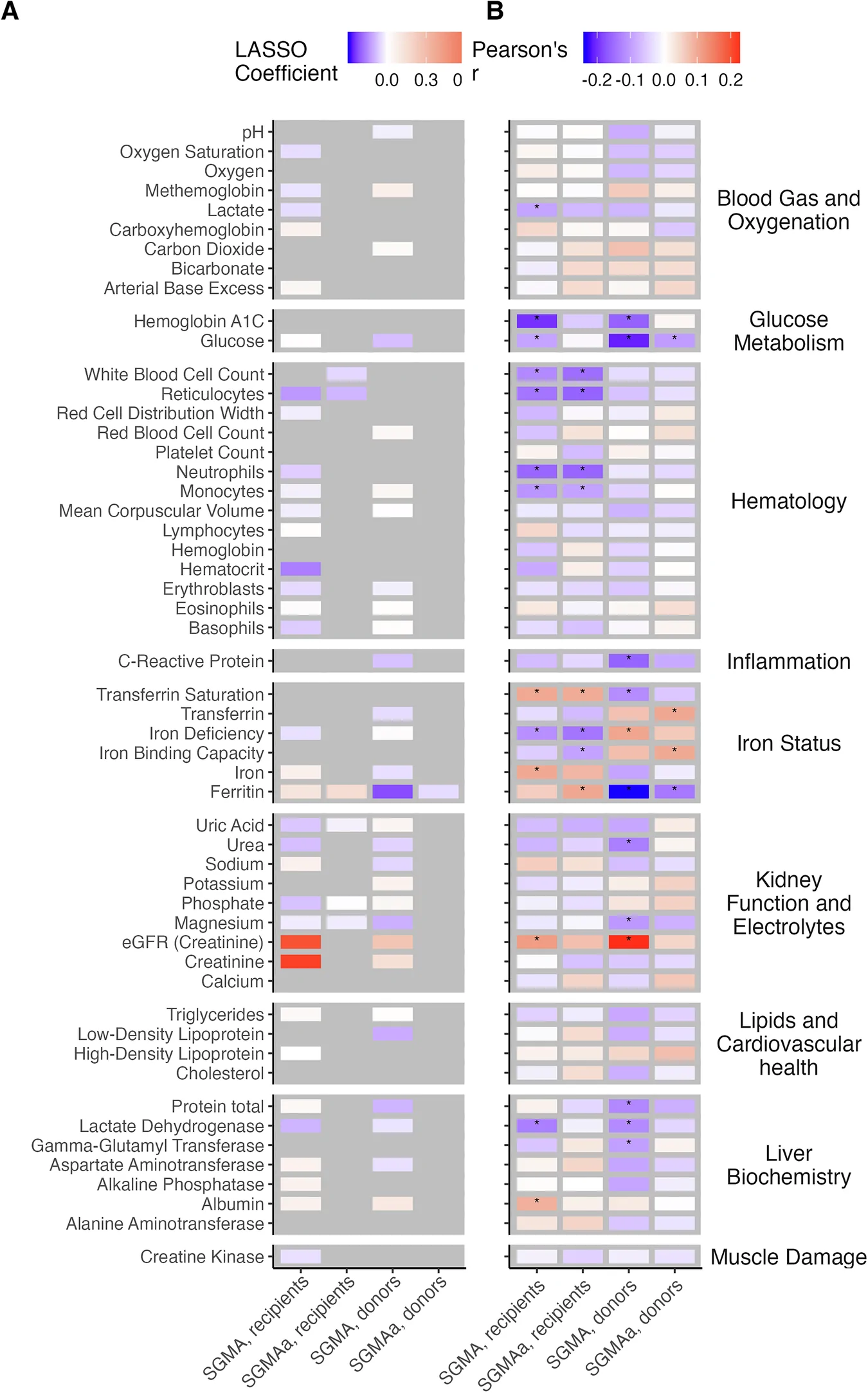
Association between sampled blood markers and memory performance. **A** (left) shows LASSO regression coefficients, for models predicting the unadjusted SGMA score for recipients, the SGMAa score for recipients, the unadjusted SGMA score for donors and the SGMAa score for donors using standardized predictors. Predictors excluded from the penalized regression (coefficients of 0) are uncolored (gray). **B** (right) shows individual Pearson’s correlation coefficients between measurements (vertical axis) and outcome measures (horizontal axis, from left to right: the unadjusted SGMA score for recipients, the SGMAa score for recipients, the unadjusted SGMA score for donors, and the SGMAa score for donors. Note that univariable associations with physiological markers (right panel) should be interpreted as exploratory and were not corrected for multiple comparisons. Stars indicate significant correlations at the level of *p* < 0.05. Sample sizes are given in Supplementary Table 4.

To assess the overall explanatory power of blood markers on memory function, we conducted four separate analyses. Given the large number of collected blood markers, we used LASSO-regression models, which apply an *L*_1_ regularization penalty to mitigate overfitting, handle multicollinearity, and perform variable selection by shrinking less informative predictors toward zero^58^. The total number of observations for the blood markers included in the analyses in shown in Supplementary Table 4. Models were conducted separately to predict (a) unadjusted SGMA scores in recipients, (b) adjusted SGMAa scores in recipients, (c) unadjusted SGMA scores in donors, and (d) adjusted SGMAa scores in donors. The left panel of Fig. 5 shows the standardized coefficients from the surviving regressors from each of these models. Remarkably, blood markers alone accounted for 36.66% and 25.52% of the variance in unadjusted SGMA scores in KTR and KD, respectively. After demographic adjustment, the variance explained remained substantial at 9.97% in recipients, but dropped to just 1.11% in donors. This demonstrates that—while the relationship between memory and physiology is partially mediated by demographic factors—physiological markers in KTR independently explain memory performance in recipients, even after accounting for age, sex, and education. The greater explained variance in KTR compared to KD likely reflects the fact that the physiological profiles were much less variable across individuals in KD compared to KTR (see Supplementary Fig. 7). In the next sections, we discuss a number of key associations of interest in more detail.

#### Biomarkers and memory function in recipients

Several key associations between blood markers and memory scores emerged among recipients, as visualized in the bottom panel of Fig. 5. For readability, we only report correlations based on the demographically adjusted scores (SGMAa) here, unless these were not statistically significant, in which case the unadjusted SGMA scores are explicitly reported. A comprehensive overview is available in Supplementary Table 5.

##### Glucose metabolism

Both glucose (*r* = − 0.09, *p* = 0.039) and glycated hemoglobin (HbA1c, *r* = − 0.20, *p* < 0.001) were negatively correlated only with the unadjusted SGMA scores, aligning with known physiological and socioeconomic demographic influences on glucose metabolism (For example, aging is linked to reduced insulin sensitivity and *β*-cell function^59^, while sex differences in hormones and body composition affect glucose regulation^60^; lower educational attainment is also associated with adverse health behaviors that are related to poorer glycemic control^61^). We did not find evidence for a link between memory function and glucose metabolism independent of these demographic mediators.

##### Hematology

Higher neutrophil (*r* = −0.16, *p* = 0.001), monocyte (*r* = − 0.09, *p* = 0.044), and white blood cell counts (*r* = −0.15, *p* = 0.001) were associated with lower SGMAa scores, suggesting that (subclinical) immune activity may be linked to reduced cognitive performance, even after demographic adjustment. Although reticulocyte count was negatively associated with SGMAa (*r* = − 0.16, *p* = 0.001), hemoglobin was not associated with SGMA(a). An explanation is that the association with hemoglobin only emerges below a certain threshold, as studies showed that anemia is associated with cognitive impairment^62^. Elevated reticulocyte count may indicate active hematopoietic stress-such as chronic blood loss, inflammation, or increased red blood cell turnover, which could cause lower memory function.

##### Iron status

Ferritin (*r* = 0.10, *p* = 0.026) and transferrin saturation were positively correlated with the SGMAa scores (*r* = 0.10, *p* = 0.034). Iron deficiency and iron binding capacity (*r* = − 0.10, *p* = 0.041) negatively correlated with the SGMAa scores (*r* = − 0.14, *p* = 0.013). Total iron levels were only significantly associated with the unadjusted SGMA scores (*r* = 0.10, *p* = 0.029; SGMAa scores: *p* = 0.066). These findings suggest that indicators of poor iron status may be linked to reduced memory performance—even after adjusting for demographic factors. This is consistent with a recent study among KTR, in which iron deficiency was associated with impaired neurocognitive tasks measuring memory, mental speed, and attention and executive functioning^63^.

##### Kidney function

eGFR correlated positively with the unadjusted SGMA scores (*r* = 0.11, *p* = 0.008), but this association disappeared after demographic adjustment. This attenuation suggests that the observed relationship was likely driven by age-, sex- or education-related differences in kidney function, rather than reflecting a direct effect of kidney function on memory performance^64^. An alternative explanation may be that by correcting for age, differences in cognitive function caused by premature decline as a result of kidney failure are adjusted for in the SGMAa, but are shown in the unadjusted SGMA.

Together, the above results demonstrate that in KTR, a substantial proportion of variance in memory performance can be explained by blood-based biomarkers alone. Notably, even after adjusting for demographic factors such as age, sex, and education, meaningful associations persist, particularly in domains related to hematologic profiles and iron status.

#### Biomarkers and memory function in (potential) donors

In line with the LASSO regression results, fewer significant associations were observed among (potential) donors, likely reflecting their more stable physiological status compared to recipients. Still, some notable patterns emerged, as described below.

##### Glucose metabolism

Higher glucose levels negatively correlated with the SGMAa scores (*r* = −0.10, *p* = 0.041).

##### Hematology and inflammation.

C-reactive protein negatively correlated with the SGMA scores (*r* = −0.16, *p* = 0.001), though this association weakened after demographic adjustment (*r* = −0.09, *p* = 0.083). Unlike in recipients, we found no associations with monocytes, neutrophils, reticulocytes, or white blood cell counts.

##### Iron status

Ferritin was negatively associated with SGMAa scores (*r* = −0.14, *p* = 0.006), while iron binding capacity showed a positive association (*r* = 0.10, *p* = 0.045). Iron deficiency positively correlated only with the unadjusted SGMA score (*r* = 0.13, *p* = 0.036), though this result should be interpreted with caution, as only 10 donors were classified as iron-deficient (see section “Demographic, Clinical, and Laboratory Variables”). Transferrin showed a negative correlation with SGMAa scores (*r* = −0.10, *p* = 0.040), transferrin saturation was negatively associated only with the unadjusted SGMA score (*r* = −0.12, *p *= 0.018). Notably, this pattern is opposite to what we observed in KTR, and reflect a complex, possibly *protective* role of mild iron deficiency in (potential) donors, aligning with prior evidence linking mild iron depletion to reduced cardiovascular risk^65^ and decreased risk of neurodegenerative diseases^66^. The divergence from the results found in KTR is not entirely surprising: in KTR iron deficiency often co-occurs with underlying disease activity or chronic inflammation. In such cases, iron-related markers can reflect ongoing systemic processes, and the typical associations may “flip”—a phenomenon commonly seen when iron homeostasis is disrupted in the context of illness.

##### Kidney function and liver biochemistry

Several associations initially significant with the unadjusted SGMA scores (eGFR: *r* = 0.23, *p* < 0.001; urea: *r* = −0.13, *p* = 0.008; magnesium: *r* = −0.11, *p* = 0.033; gamma-GT: *r* = −0.10, *p* = 0.047; LDH: *r* = −0.12, *p* = 0.017) lost significance after demographic adjustment.

The fewer and weaker associations observed in KD compared to KTR are both expected and informative, reflecting the donors’ generally healthier and more stable physiological profiles. In addition, several patterns (e.g., associations with glucose, glycated hemoglobin, and eGFR, although mediated by age, sex, or education level) mirrored the associations found in recipients. This consistency between groups strengthens confidence in the relevance of these physiological factors for memory performance, even in populations with minimal clinical burden.

An important consideration in interpreting these results is the temporal gap between the laboratory measurements and the memory assessments. In our sample, the median interval was 2.28 years (*IQR* = 0.73−4.72 years), meaning that physiological parameters and cognitive outcomes were not always assessed concurrently. Supplementary Fig. 8 shows that, when stratifying the sample by time since laboratory assessment, correlation patterns between blood markers and SGMA scores were broadly consistent across subgroups. Nonetheless, replication of these analyses with *concurrent* laboratory and SGMA data would be valuable, as it would allow for more precise estimation of short-term physiological influences on memory performance and reduce the potential for temporal confounding.

## Discussion

This study had two primary objectives: to evaluate the feasibility and psychometric performance of the SGMA as a memory screening tool, and to examine how transplant-related clinical and physiological factors relate to memory function in this population. Together, the findings position SGMA as a practical instrument for remote cognitive assessment while offering new insight into the cognitive phenotype associated with kidney transplantation.

A practical cognitive screening tool should be accessible (low-burden, time-efficient, and suitable for remote use), reliable (yielding stable results over repeated measures), and valid (consistently capturing a distinct cognitive construct). Overall, our findings suggest that the SGMA meets these criteria in a clinical context.

First, our findings indicate that the SGMA is feasible for use in this population. High engagement across sessions suggests that participants were willing and able to complete repeated memory assessments using the remote digital platform, even in the presence of complex chronic health conditions.

Second, the observed stability of SGMA scores across repeated assessments suggests that the instrument is well-suited for tracking cognitive change longitudinally. The absence of clear practice effects indicates that the SGMA captures relatively stable aspects of memory function rather than effects of task familiarity.

Third, the finding that SGMA scores were related to established neuropsychological measures of memory, attention, and processing speed suggests that the instrument captures core cognitive processes relevant to everyday functioning. The presence of these associations despite a substantial temporal gap between assessments indicates that the SGMA may reflect stable individual differences in cognitive function. These relationships therefore likely underestimate the strength of associations that would be observed with concurrent benchmarkings.

Given the above, the SGMA addresses several key limitations of conventional cognitive screening tools (e.g., MoCA, MMSE). While such instruments are widely adopted in clinical settings, their nonadaptive nature often has a negative impact on the test-taker experience. Moreover, substantial test-retest effects limit the reliability of these screening tools in longitudinal contexts^14^. The SGMA accommodates these challenges by offering an accessible alternative that maintains reliability and validity, making it well-suited for repeated cognitive screening in clinical settings. It is good to note that successful completion of the SGMA (like the MoCA, CERAD, and MMSE) does presuppose basic literacy, which may limit its applicability in some populations.

In the second part of this study, our objective was to evaluate the memory function in KTR by comparing their performance to that of (potential) KD and examining associations with clinical variables, including (pre)transplant factors and time since surgery.

Our finding that memory performance was lower in KTR than in (potential) KD after demographic adjustment suggests a modest but persistent cognitive disadvantage compared with a demographically similar, generally healthy population. At present, minimal clinically important differences (MCIDs) for the SGMA have not yet been established, reflecting the recent development of the instrument. To aid interpretation of the magnitude of this effect, the observed group difference can be contextualized using the association between age and SGMA performance in the current sample, which—based on the observed age slope—corresponds to an estimated “memory age” difference of roughly 5–6 years. Prior work using the SGMA has reported differences of approximately 20 SGMA points between individuals with mild cognitive impairment and healthy controls^19^ suggesting that the effect observed here is modest in magnitude relative to differences observed when comparing clinical groups.

The absence of associations with time since transplantation, dialysis history, or donor type suggests that the observed cognitive differences are not driven solely by post-transplant progression. These findings align with prior work indicating persistent cognitive differences after transplantation. In the TransplantLines Biobank and Cohort study, 16% of KTR met criteria for mild cognitive impairment compared with 2.6% of KD, with no associations observed for time since transplantation, dialysis history, or donor type^2^. Similarly, Gupta et al.^7^ showed that semantic memory function does not fully recover following KT. Together, this suggests that cognitive deficits in recipients may persist after transplantation and reflect broader, lasting effects of kidney failure and/or its treatment. It is good to note that the results of the current study are based on cross-sectional data, and we were unable to examine longitudinal trajectories of kidney function (e.g., pre- to post-transplant or donation changes in eGFR), which may be more informative for cognitive outcomes than time since transplantation alone. Future work incorporating repeated SGMA assessments alongside concurrent renal function measurements would help clarify these temporal relationships. In addition, future research is needed to explore the underlying mechanisms driving the lasting differences between KTR and controls following transplantation, including the potential impact of immunosuppressive medication^67–69^, comorbidities and the clinical course early post-transplantation.

The observed associations between memory performance and sleep quality, fatigue, and general health suggest that day-to-day well-being is meaningfully related to cognitive functioning, although the effect sizes were modest. Our results are in line with established mechanisms such as reduced attentional resources following sleep disruption and fatigue^70^, impaired sleep-dependent memory consolidation^71^ and mechanisms linking broader physiological burden to brain systems supporting memory^72,73^. These findings underscore that cognitive performance in this population is shaped not only by clinical and biological factors, but also by subjective health and daily functioning.

Our analysis of blood markers provided additional insight into the physiological correlates of memory function. Among recipients, lower eGFR, elevated reticulocyte counts, higher ferritin, glucose, and glycated hemoglobin levels, as well as increased monocyte and neutrophil counts, were associated with poorer memory performance. While some associations were attenuated after adjusting for demographic variables, several remained robust, particularly those related to iron status and hematology. Among (potential) donors, the number and strength of the associations were generally lower, consistent with the donors’ more stable health status and narrower physiological variability. Because no formal multiple-testing correction was applied to the univariable biomarker analyses, individual associations should be interpreted with caution and should be considered hypothesis-generating rather than confirmatory. The complementary use of LASSO regression partly mitigates this risk by applying shrinkage and variable selection, providing a more conservative estimate of the relative contribution of physiological markers to memory performance.

This study has a number of limitations. First, its cross-sectional design precludes conclusions about the directionality or causality of the observed associations between physiological markers and memory performance. In addition, blood samples and SGMA sessions were not completed concurrently (median interval: 2.26 years), which likely attenuated the strength of associations; the same applies to the neuropsychological assessments conducted years earlier. Although analyses suggested that these relationships were relatively robust to measurement timing (see Supplementary Figs. 3 and 8), future work with concurrent assessments is needed.

Another limitation concerns the exclusion of sessions based on predefined performance thresholds to ensure reliable estimation of the forgetting parameter. While these criteria were intentionally lenient and primarily aimed at identifying disengagement or interruptions rather than poor memory performance, they may have occurred more often among KTR, potentially leading to a conservative estimate of group differences. Finally, comorbidities were not included as covariates in the primary analyses, as the aim was to capture effects related to the overall clinical phenotype of KTR; however, this may have introduced residual confounding and should be considered when interpreting the findings. Future research should employ a longitudinal design with repeated SGMA sessions and concurrent biomarker collection to more accurately study temporal dynamics and potentially causal pathways linking systemic health to cognitive change. SGMA uniquely allows for this requirement of repeated testing. This study demonstrates the potential of computational memory assessment as a feasible, accurate, and scalable tool for cognitive screening in complex clinical populations. The SGMA combines a patient-friendly design with good psychometric properties and sensitivity to physiological burden, making it ideal for both scientific research and clinical monitoring.

In clinical settings, the SGMA offers significant opportunities for scalable cognitive monitoring, particularly in populations at elevated risk of cognitive impairment. KTR are known to face increased risk of developing mild cognitive impairment and Alzheimer’s disease, yet cognitive assessment is not routinely implemented in their standard care. Our findings demonstrate that the SGMA is not only feasible in this population but also shows good reliability and validity. These properties position the SGMA as a viable tool for regular, remote monitoring of cognitive status. The results of this screening could lead to further neuropsychological investigation and subsequently facilitate earlier identification of decline and support targeted interventions such as cognitive rehabilitation or medication adherence support. More broadly, our results suggest that the SGMA captures clinically meaningful variance in cognitive function, even after demographic adjustment, reinforcing its potential role in personalized care strategies and long-term outcome monitoring in individuals with complex health conditions.

## Supplementary information

Supplementary Materials

Description of Additional Supplementary files

Supplementary Data File 1

Supplementary Data File 2

Supplementary Data File 3

Supplementary Data File 4

Supplementary Data File 5

Supplementary Data File 6

## Data Availability

SGMA data are publicly available at https://osf.io/p7aus/. Due to patient confidentiality, the clinical data associated with the SGMA dataset are not publicly available but can be made available upon request. Access to this clinical dataset requires a minimal access procedure consisting of a request by email (datarequest.transplantlines@umcg.nl). A response will be provided within 2 weeks. This access procedure is to ensure that the clinical data are being requested for research/scientific purposes only and thus complies with the informed consent signed by TransplantLines participants, which specifies that the collected data will not be used by commercial parties. Source data to generate the main figures in this manuscript is available in the Supplementary Data Files: Supplementary Data File 1 for Fig. 1, Supplementary Data File 2 for Fig. 2, Supplementary Data File 3 for Fig. 3, Supplementary Data File 4 for Fig. 4, Supplementary Data File 5 for Fig. 5a, and Supplementary Data File 6 for Fig. 5b.

## Code availability

Analysis code is publicly available at https://osf.io/p7aus/.
