# Supplementary material for: Computational assessment of memory function in kidney transplant recipients and donors
Source: Commun Med (Lond). 2026 Jun 5;6:480. doi: 10.1038/s43856-026-01663-x (PMC13562709; doi:10.1038/s43856-026-01663-x)
Supplement: Supplementary file 1 [file 43856_2026_1663_MOESM1_ESM.pdf]

Computational Assessment of Memory Function in Kidney Transplant Recipients and Donors: Supplementary materials

Flow diagram

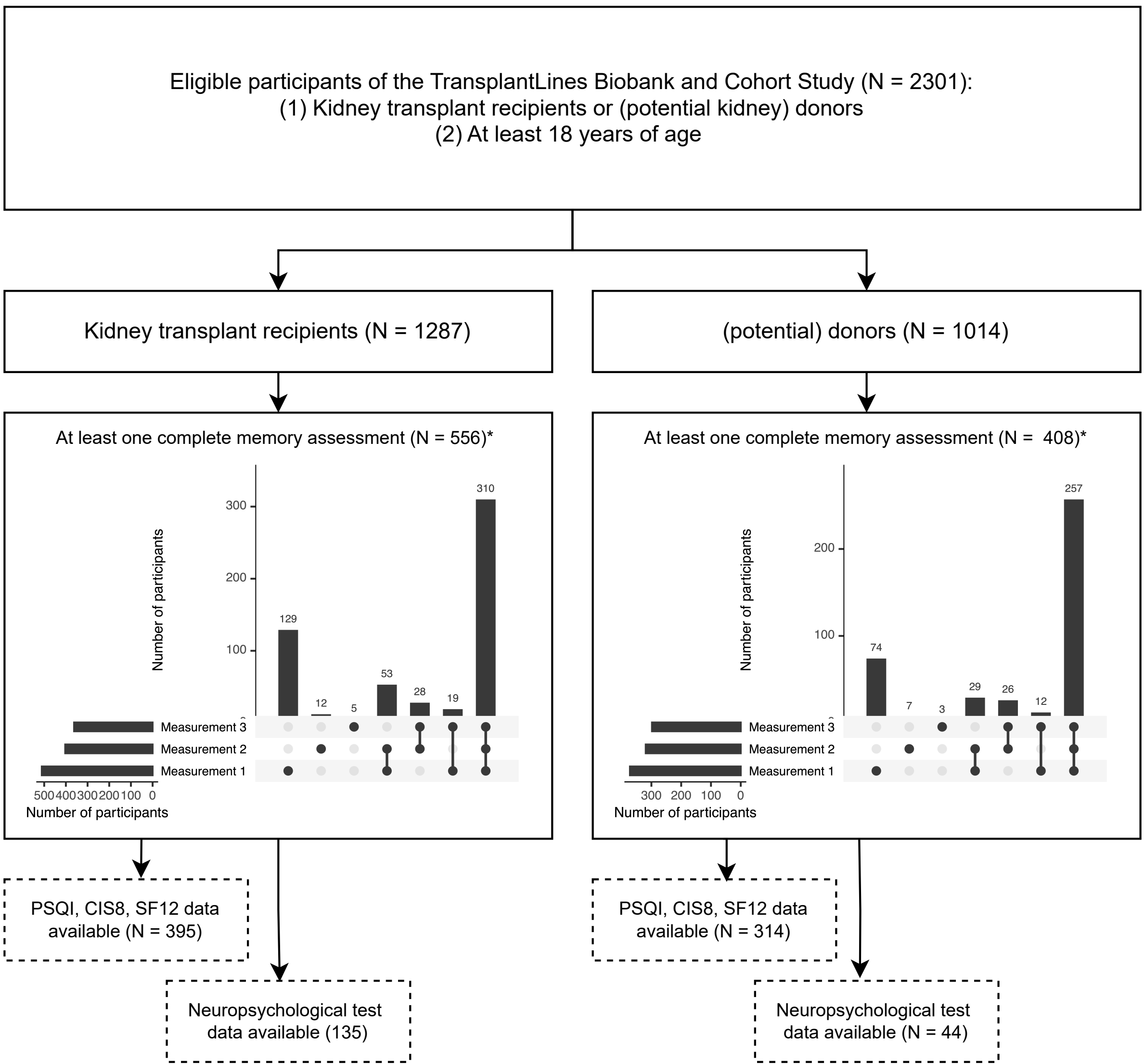

*All participants with at least one complete memory assessment were included in the study.

Supplementary Figure 1. Flow diagram for participant inclusion, including upset plots for the three memory measurements in this study.

Study Timeline

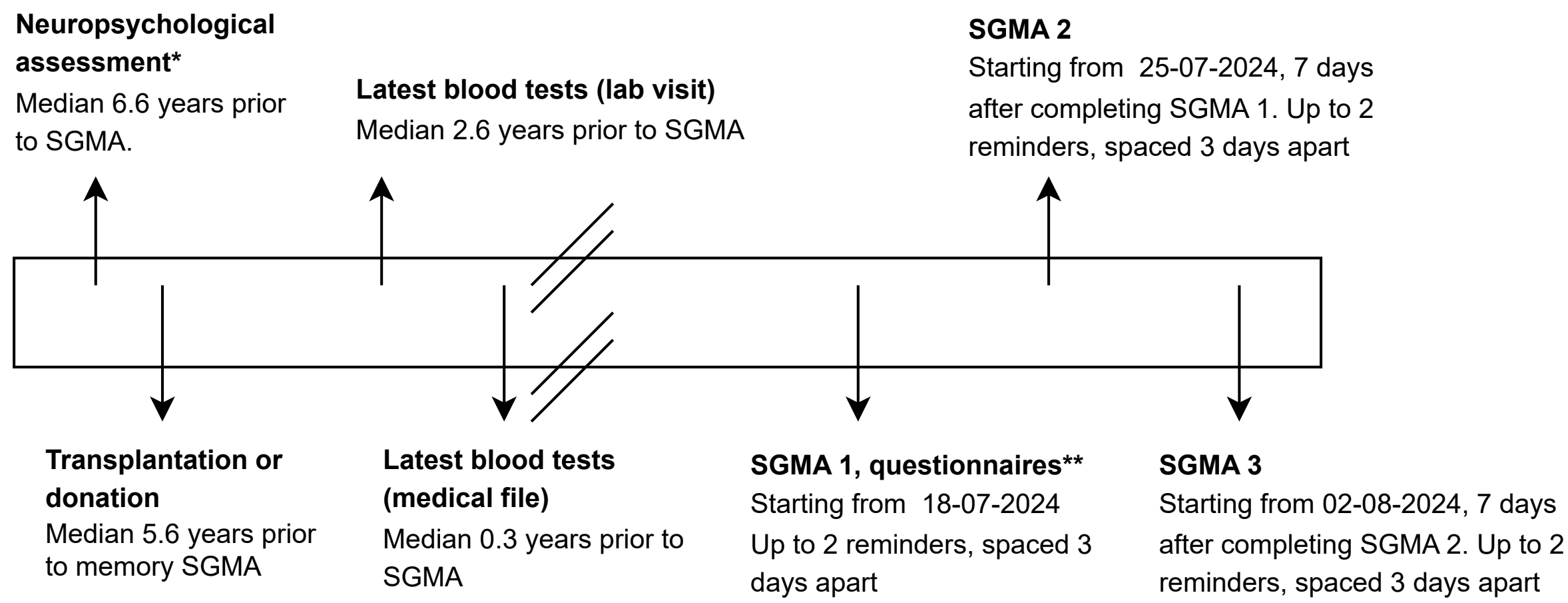

Supplementary Figure 2. Timeline for key study time points. *Neuropsychological assessments were completed exclusively between 2015–2021. **Questionnaires included basic demographics, patient-reported outcome measures (CIS8R, SF12, PSQI), and ratings of subjective memory score.

Sample characteristics for responders and non-responders

Supplementary Table 1. Sample characteristics for non-responders and responders. For time since donation or transplantation the median (IQR) value is shown, the other rows show means (SD) or absolute numbers and proportions. Differences between responders and non-responders were assessed using two-sided Welch two-sample t-tests.

	**Not responded**	**Responded**	*p*
Recipients			
N	731	556	
Age (years)	58.37 (14.28)	58.59 (12.64)	0.77
N female	263 (36%)	222 (40%)	0.18
Years since transplantation	6.40 (2.50–11.32)	6.06 (3.10–10.94)	0.94
N pre-transplant dialysis	446 (61%)	311 (56%)	0.08
N living donor	402 (55%)	344 (62%)	**0.01**
eGFR (mL/min/1.73 m²)*	52.47 (18.10)	53.16 (17.53)	0.50
Donors/potential donors			
N	606	408	
Age (years)	60.58 (12.21)	63.27 (10.77)	< **0.001**
N female	303 (50%)	228 (56%)	0.09
Years since donation	5.76 (2.76–7.83)	4.98 (2.21–7.72)	0.26
eGFR* (mL/min/1.73 m²)	68.57 (16.92)	63.09 (13.80)	< **0.001**

**estimated glomerular filtration rate (creatine).*

Neuropsychological tests for recipients and donors

Supplementary Table 2. Means and standard deviations of neuropsychological tests for recipients and donors. Differences between recipients and donors were assessed using two-sided Welch two-sample t-tests.

Test	Recipient	Donor	*p*	Domain
Trail Making Test-B	66.85 (25.62)	60.42 (21.12)	0.10	Attention
Clock Drawing	11.89 (1.80)	11.91 (1.41)	0.94	Executive Function
Dutch Reading Test	13.79 (0.42)	13.88 (0.27)	0.10	Language
Word fluency (animals)	24.02 (5.53)	28.07 (5.36)	< **0.001**	Language
Word fluency (letters)	35.26 (12.11)	42.95 (11.40)	< **0.001**	Language
Word fluency (professions)	16.68 (3.97)	20.42 (4.58)	< **0.001**	Language
15 Words Test - Delayed retention	9.58 (2.96)	9.17 (3.19)	0.47	Memory
15 Words Test - Immediate recall	43.44 (9.51)	42.41 (10.73)	0.59	Memory
Digit Span Backwards	8.69 (1.86)	9.14 (1.98)	0.19	Memory
Digit Span Forwards	7.88 (2.13)	8.14 (1.76)	0.43	Memory
Symbol Digits Modalities	49.81 (10.01)	50.67 (8.43)	0.58	Processing Speed
Trail Making Test-A	30.88 (11.50)	30.03 (10.72)	0.66	Processing Speed

Time since neuropsychological assessment

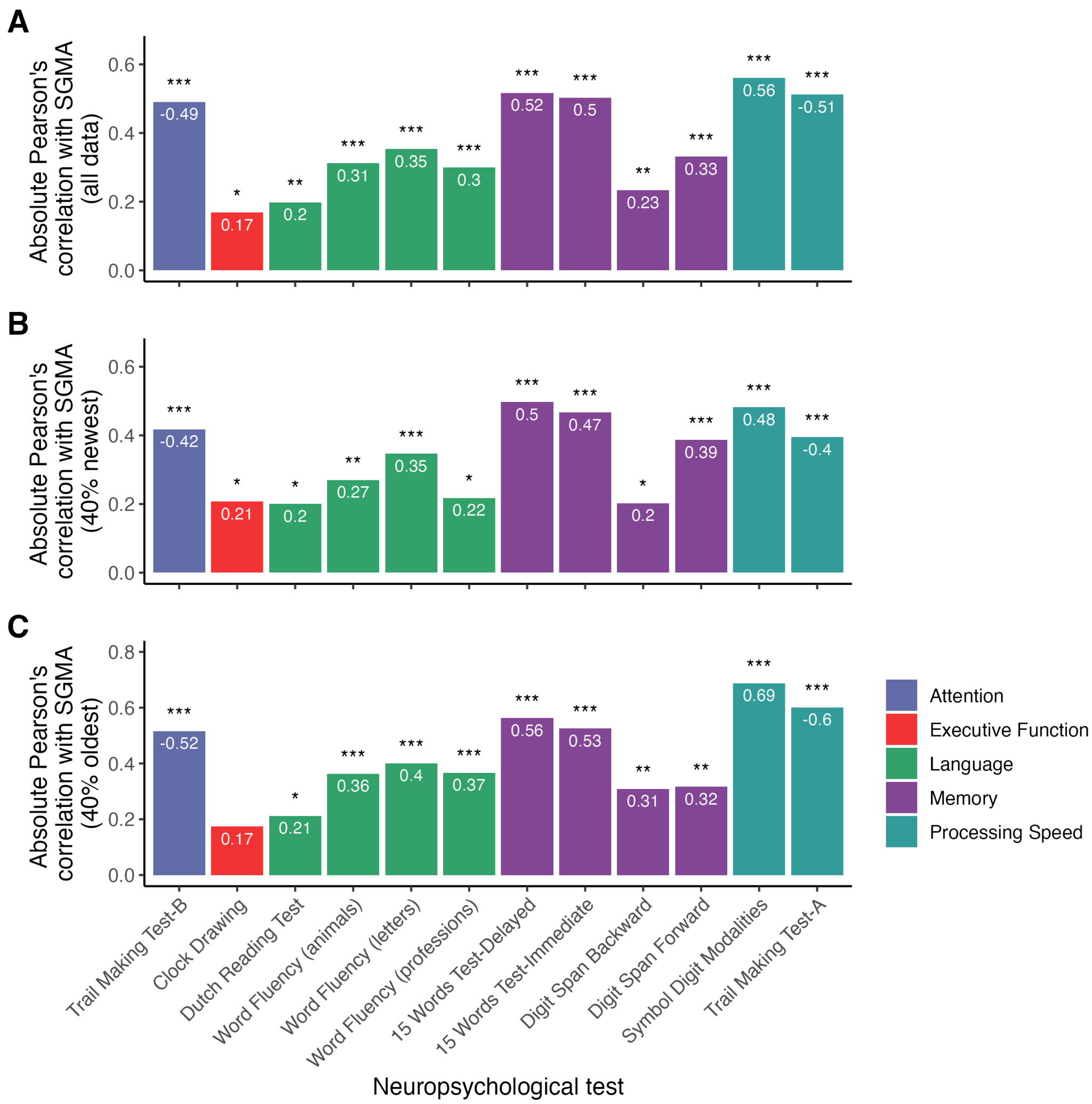

Supplementary Figure 3. **Panel A** shows absolute Pearson's correlations between SGMA scores and a range of neuropsychological tests across the full dataset (corresponding to main Figure 3). **Panel B** shows correlations restricted to participants with the most recent neuropsychological assessment (40% most recent observations). **Panel C** shows correlations restricted to participants with the oldest assessments (40% oldest observations). Bars are color-coded by cognitive domain. $*** : p < 0.001$; $** : p < 0.01$; $* : p < 0.05$. n = 179 participants.

Subjective memory ratings

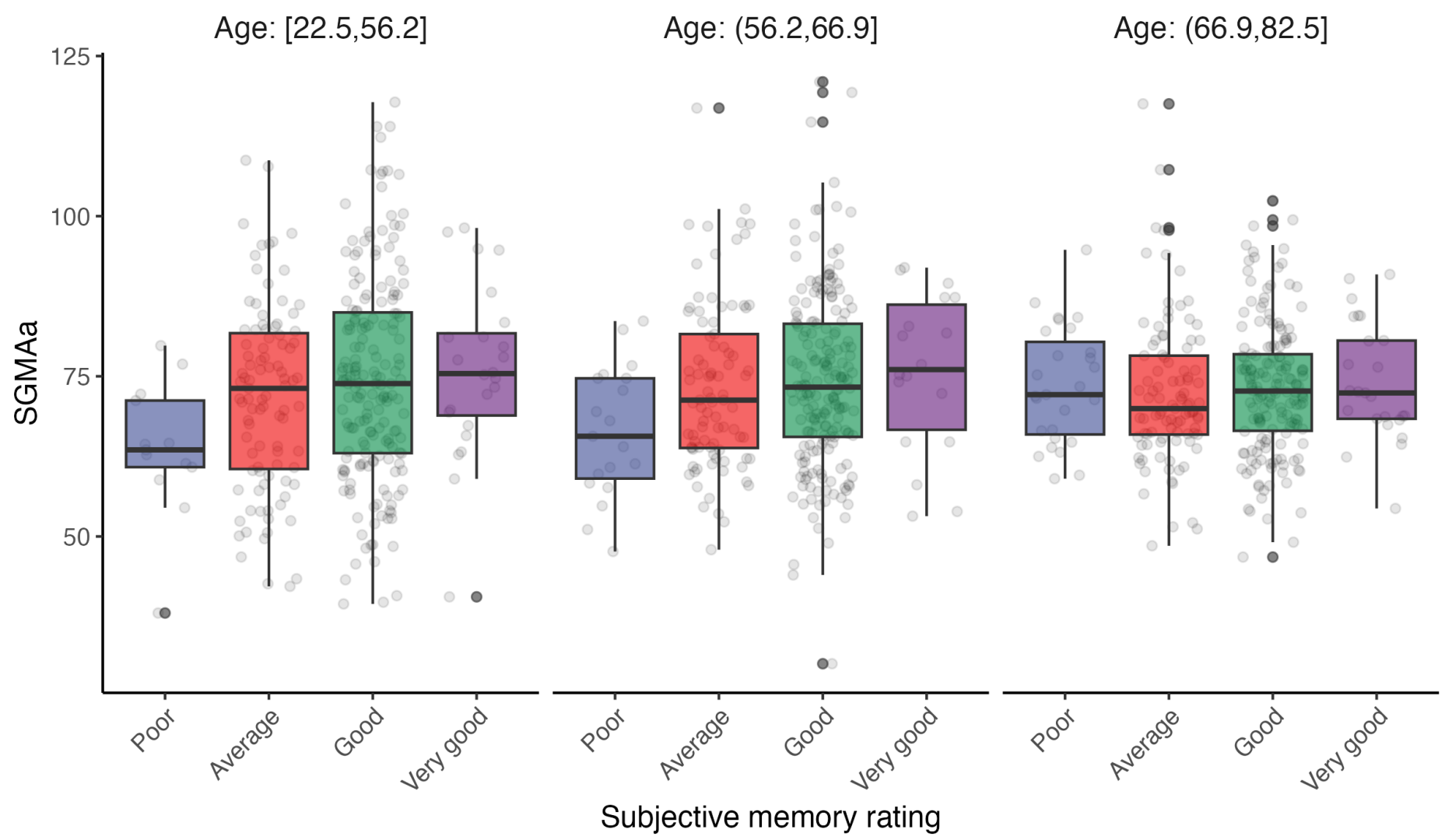

Supplementary Figure 4. Relationship between objective memory performance as measured by the SGMA, adjusted for demographics, and subjective ratings of memory performance. Participants are divided into three equally-sized age brackets: between 22.5–56.2 years, between 56.2–66.9 years and between 66.9–82.5 years. While in the younger and middle-age participants, there was an association between subjective and objective memory, this relationship was not found in elderly participants. n = 956 participants.

Sensitivity analysis

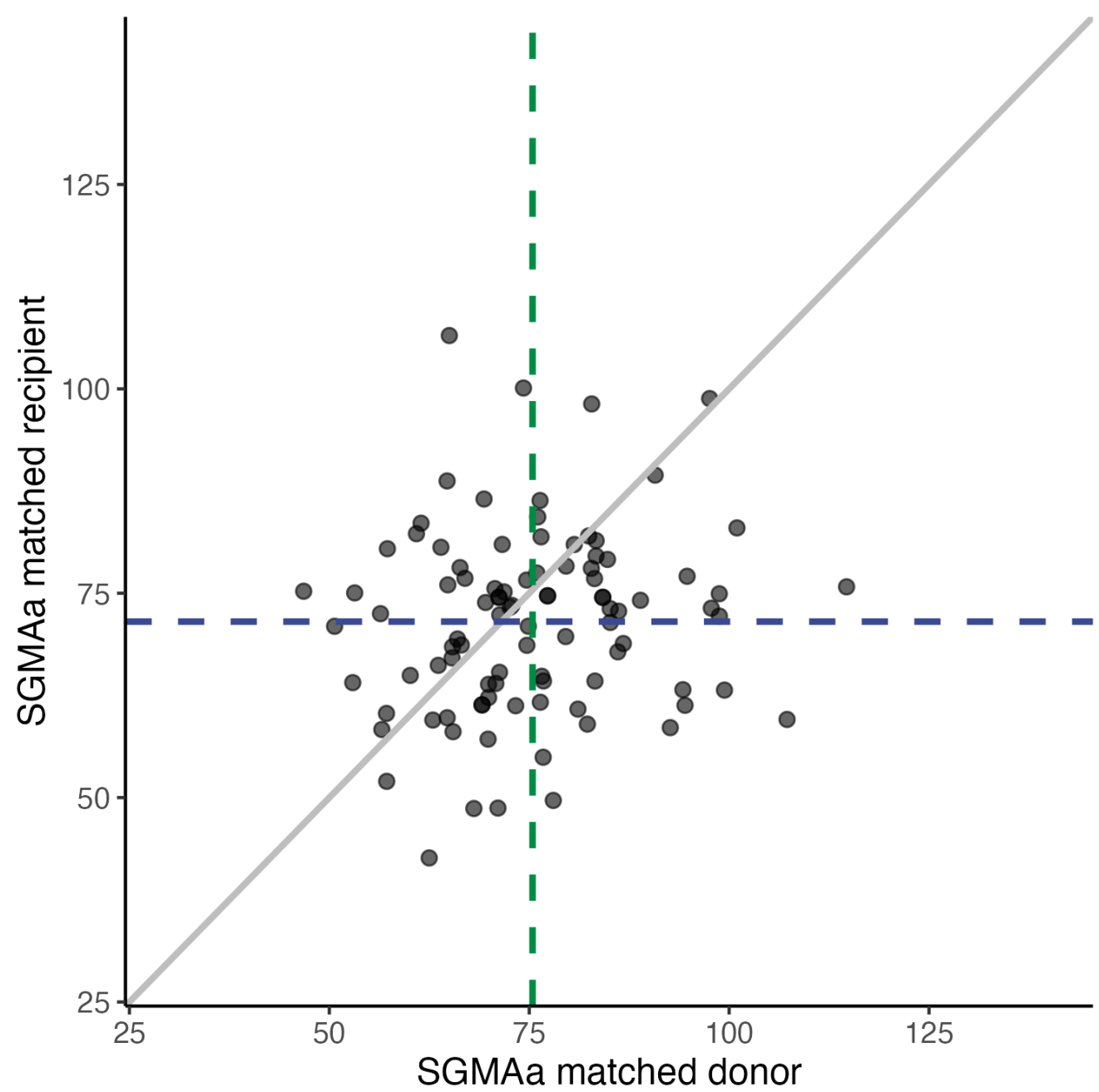

Supplementary Figure 5. Scatterplot of SGMAa scores in KTR versus SGMAa scores of their donors. Each dot represents one matched pair. The solid diagonal line indicates equal performance between groups, while dashed lines show mean SGMAa scores for donors (green) and recipients (blue). Even under matched conditions, recipients scored lower on SGMAa than donors, confirming reduced memory performance in KTR. n = 96 matched pairs.

Primary kidney disease

Supplementary Table 3. Numbers indicate p-values for pairwise two-sided Welch t-tests of SGMAa scores of KTR with etiologies of kidney failure.

	Cystic KD	Glomerulo-nephritis	Interstitial-nephritis	Other congenital and hereditary KD	Renal vascular disease, excluding vasculitis	Diabetes Mellitus	Other multisystem diseases	Unknown
Glomerulonephritis	0.09							
Interstitial-nephritis	0.07	0.53						
Other congenital and hereditary KD	0.62	0.66	0.41					
Renal vascular disease, excluding vasculitis	0.78	0.32	0.19	0.80				
Diabetes Mellitus	**0.03**	0.24	0.59	0.22	0.08			
Other multisystem diseases	0.38	0.75	0.44	0.87	0.61	0.21		
Unknown	0.11	0.88	0.65	0.61	0.31	0.32	0.68	
Other	0.44	0.96	0.67	0.78	0.58	0.40	0.87	0.90

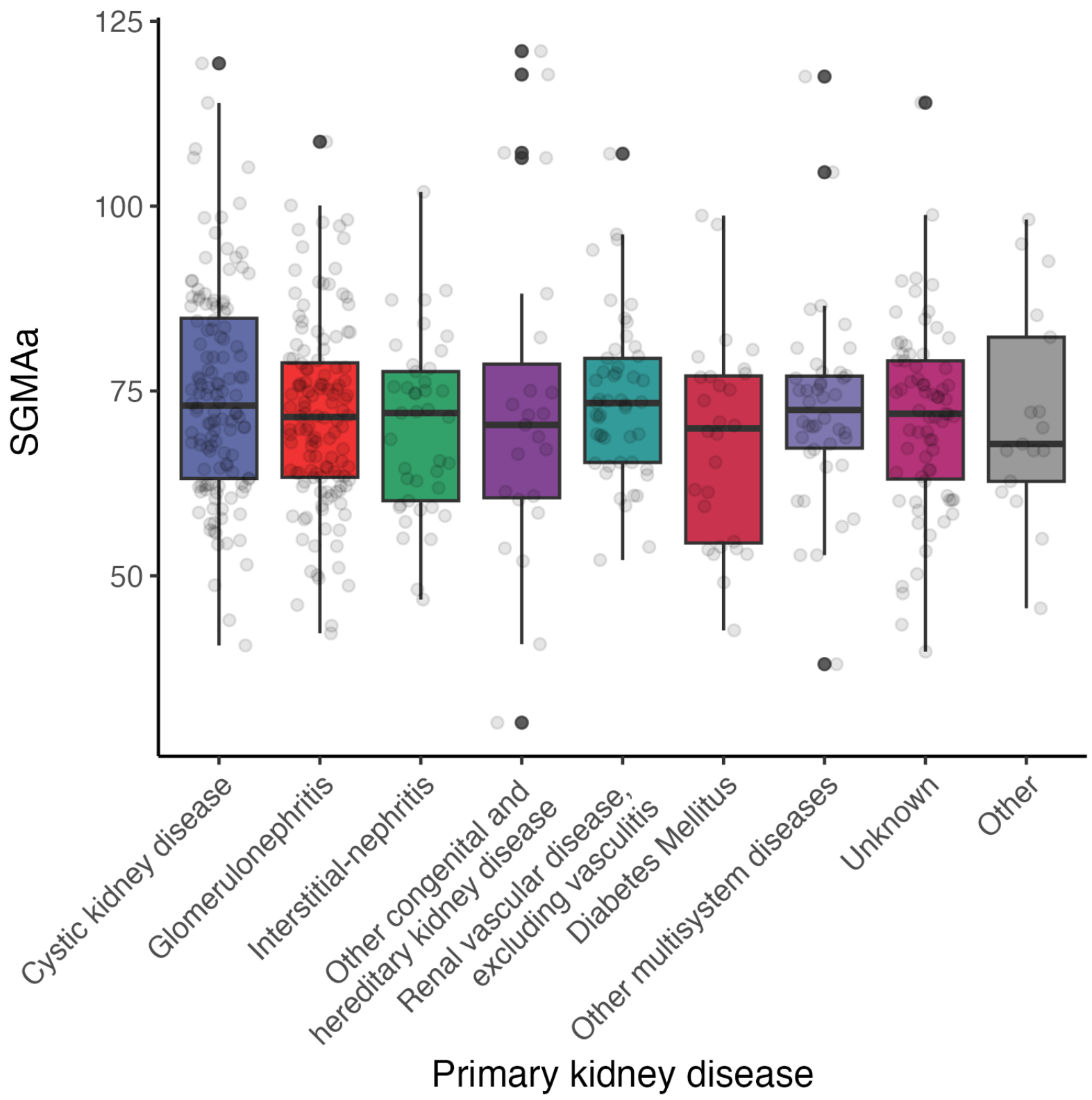

Supplementary Figure 6. SGMAa values for KTR stratified by primary kidney disease etiology. Group sizes were: cystic kidney disease (n = 128), glomerulonephritis (n = 128), unknown (n = 100), renal vascular disease excluding vasculitis (n = 49), other multisystem diseases (n = 42), interstitial nephritis (n = 41), diabetes mellitus (n = 28), other congenital and hereditary kidney disease (n = 23), and other (n = 17).

Lab marker variance

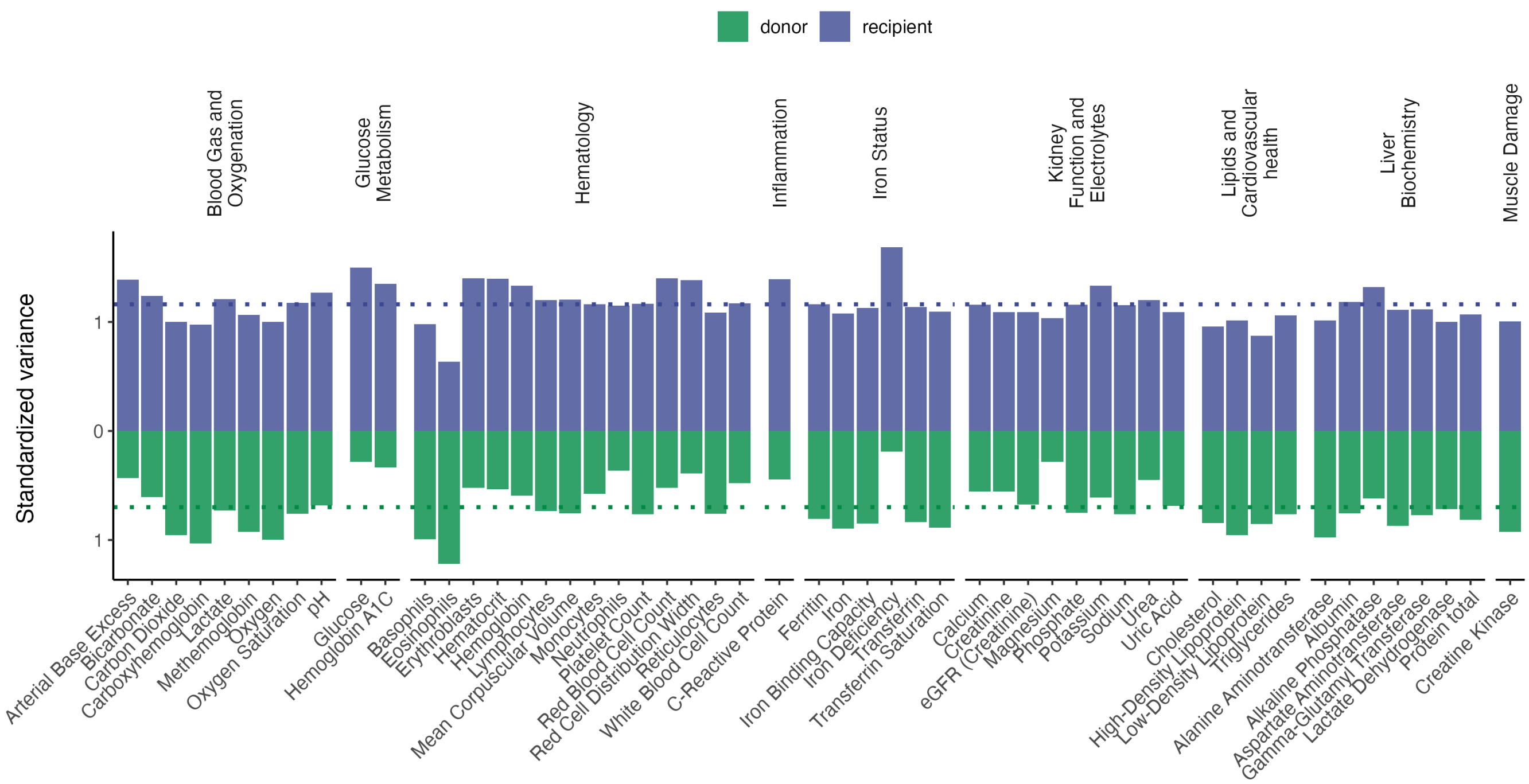

Supplementary Figure 7. Average standardized variance by lab measurement, separated for recipients (blue, plotted upwards) and (potential) donors (green, plotted downwards). Dotted lines show averages across markers.

Blood markers: available observations

Supplementary Table 4. Total number of available observations per included lab measurement.

	Blood marker	Category	Available observations (KD)	Available observations (KTR)
1	Arterial Base Excess	Blood Gas and Oxygenation	396	452
2	Bicarbonate	Blood Gas and Oxygenation	396	452
3	Carbon Dioxide	Blood Gas and Oxygenation	396	452
4	Carboxyhemoglobin	Blood Gas and Oxygenation	396	451
5	Lactate	Blood Gas and Oxygenation	393	445
6	Methemoglobin	Blood Gas and Oxygenation	396	451
7	Oxygen	Blood Gas and Oxygenation	396	451
8	Oxygen Saturation	Blood Gas and Oxygenation	396	452
9	pH	Blood Gas and Oxygenation	396	452
10	Glucose	Glucose Metabolism	405	481
11	Hemoglobin A1C	Glucose Metabolism	407	479
12	Basophils	Hematology	394	450
13	Eosinophils	Hematology	394	450
14	Erythroblasts	Hematology	401	458
15	Hematocrit	Hematology	407	479
16	Hemoglobin	Hematology	408	483
17	Lymphocytes	Hematology	394	450
18	Mean Corpuscular Volume	Hematology	407	479
19	Monocytes	Hematology	394	450
20	Neutrophils	Hematology	394	450
21	Platelet Count	Hematology	407	480
22	Red Blood Cell Count	Hematology	404	479
23	Red Cell Distribution Width	Hematology	404	471
24	Reticulocytes	Hematology	407	465
25	White Blood Cell Count	Hematology	407	484
26	C-Reactive Protein	Inflammation	408	494
27	Ferritin	Iron Status	402	471
28	Iron	Iron Status	402	472
29	Iron Binding Capacity	Iron Status	402	462
30	Iron Deficiency	Iron Status	402	459
31	Transferrin	Iron Status	402	471
32	Transferrin Saturation	Iron Status	402	444
33	Calcium	Kidney Function and Electrolytes	408	481
34	Creatinine	Kidney Function and Electrolytes	408	532
35	Magnesium	Kidney Function and Electrolytes	406	480
36	Phosphate	Kidney Function and Electrolytes	408	481
37	Potassium	Kidney Function and Electrolytes	408	481
38	Sodium	Kidney Function and Electrolytes	408	533
39	Urea	Kidney Function and Electrolytes	408	488
40	Uric Acid	Kidney Function and Electrolytes	406	468
41	eGFR (Creatinine)	Kidney Function and Electrolytes	379	530
42	Cholesterol	Lipids and Cardiovascular health	408	481
43	High-Density Lipoprotein	Lipids and Cardiovascular health	408	481
44	Low-Density Lipoprotein	Lipids and Cardiovascular health	408	481
45	Triglycerides	Lipids and Cardiovascular health	408	481
46	Alanine Aminotransferase	Liver Biochemistry	408	481
47	Albumin	Liver Biochemistry	408	531
48	Alkaline Phosphatase	Liver Biochemistry	408	480
49	Aspartate Aminotransferase	Liver Biochemistry	408	481
50	Gamma-Glutamyl Transferase	Liver Biochemistry	408	480
51	Lactate Dehydrogenase	Liver Biochemistry	404	475
52	Protein total	Liver Biochemistry	408	474
53	Creatine Kinase	Muscle Damage	404	473

Blood marker correlations

Supplementary Table 5. Correlation coefficients for the association between lab values and memory score, stratified by category and outcome measure. Values between brackets indicate p-values. Note that p-values were not corrected for multiple comparisons.

	Blood marker	Category	SGMA, all	SGMAa, all	SGMA, recipients	SGMAa, recipients	SGMA, donors	SGMAa, donors
1	Arterial Base Excess	Blood Gas and Oxygenation	-0.01 (0.878)	0.06 (0.087)	-0.01 (0.892)	0.04 (0.344)	0.01 (0.835)	0.05 (0.347)
2	Bicarbonate	Blood Gas and Oxygenation	0 (0.990)	0.06 (0.090)	-0.02 (0.706)	0.05 (0.334)	0.04 (0.387)	0.04 (0.406)
3	Carbon Dioxide	Blood Gas and Oxygenation	0.02 (0.504)	0.05 (0.176)	-0.01 (0.843)	0.04 (0.447)	0.07 (0.150)	0.04 (0.436)
4	Carboxyhemoglobin	Blood Gas and Oxygenation	0.03 (0.396)	-0.02 (0.591)	0.05 (0.324)	0.01 (0.846)	0.01 (0.869)	-0.06 (0.273)
5	Lactate	Blood Gas and Oxygenation	-0.08 (0.016)	-0.06 (0.074)	-0.09 (0.049)	-0.07 (0.132)	-0.07 (0.140)	-0.02 (0.630)
6	Methemoglobin	Blood Gas and Oxygenation	0.03 (0.422)	0 (0.896)	0 (0.976)	0 (0.950)	0.06 (0.215)	0.02 (0.673)
7	Oxygen	Blood Gas and Oxygenation	-0.02 (0.577)	-0.01 (0.699)	0.02 (0.636)	0.01 (0.891)	-0.07 (0.153)	-0.04 (0.387)
8	Oxygen Saturation	Blood Gas and Oxygenation	-0.02 (0.551)	-0.01 (0.726)	0.01 (0.802)	0 (0.953)	-0.07 (0.183)	-0.05 (0.313)
9	pH	Blood Gas and Oxygenation	-0.03 (0.332)	0 (0.963)	0 (0.945)	0 (0.958)	-0.08 (0.096)	-0.01 (0.804)
10	Glucose	Glucose Metabolism	-0.11 (0.001)	-0.05 (0.152)	-0.09 (0.039)	-0.01 (0.872)	-0.21 (0.000)	-0.10 (0.041)
11	Hemoglobin A1C	Glucose Metabolism	-0.17 (0.000)	-0.06 (0.067)	-0.20 (0.000)	-0.05 (0.271)	-0.17 (0.001)	0 (0.987)
12	Basophils	Hematology	-0.02 (0.491)	-0.02 (0.598)	-0.04 (0.440)	-0.06 (0.192)	-0.01 (0.885)	0.01 (0.777)
13	Eosinophils	Hematology	0.02 (0.627)	0.04 (0.270)	0.03 (0.551)	-0.01 (0.825)	0.01 (0.845)	0.04 (0.422)
14	Erythroblasts	Hematology	-0.04 (0.259)	-0.04 (0.274)	-0.03 (0.501)	-0.04 (0.379)	-0.06 (0.258)	-0.01 (0.905)
15	Hematocrit	Hematology	-0.07 (0.029)	0.01 (0.692)	-0.09 (0.057)	0.02 (0.639)	-0.05 (0.325)	0 (0.973)
16	Hemoglobin	Hematology	-0.05 (0.105)	0.02 (0.482)	-0.06 (0.188)	0.03 (0.558)	-0.04 (0.370)	0 (0.949)
17	Lymphocytes	Hematology	0.02 (0.520)	-0.02 (0.604)	0.05 (0.304)	-0.04 (0.453)	-0.02 (0.689)	-0.02 (0.738)
18	Mean Corpuscular Volume	Hematology	-0.04 (0.197)	-0.03 (0.363)	-0.02 (0.622)	-0.03 (0.52)	-0.08 (0.118)	-0.04 (0.379)
19	Monocytes	Hematology	-0.08 (0.018)	-0.08 (0.019)	-0.11 (0.020)	-0.09 (0.044)	-0.05 (0.350)	0 (0.963)
20	Neutrophils	Hematology	-0.10 (0.004)	-0.14 (0.000)	-0.16 (0.001)	-0.16 (0.001)	-0.02 (0.635)	-0.04 (0.431)
21	Platelet Count	Hematology	0.01 (0.665)	-0.03 (0.329)	0.01 (0.743)	-0.07 (0.133)	0.02 (0.745)	-0.01 (0.882)
22	Red Blood Cell Count	Hematology	-0.04 (0.221)	0.03 (0.336)	-0.06 (0.168)	0.04 (0.441)	0 (0.951)	0.04 (0.435)
23	Red Cell Distribution Width	Hematology	-0.05 (0.145)	-0.02 (0.582)	-0.07 (0.111)	-0.01 (0.879)	-0.02 (0.750)	0.03 (0.590)
24	Reticulocytes	Hematology	-0.10 (0.002)	-0.12 (0.000)	-0.14 (0.002)	-0.16 (0.001)	-0.06 (0.208)	-0.03 (0.513)
25	White Blood Cell Count	Hematology	-0.08 (0.015)	-0.13 (0.000)	-0.12 (0.008)	-0.15 (0.001)	-0.04 (0.478)	-0.03 (0.519)
26	C-Reactive Protein	Inflammation	-0.09 (0.007)	-0.07 (0.039)	-0.07 (0.128)	-0.04 (0.348)	-0.16 (0.001)	-0.09 (0.083)
27	Ferritin	Iron Status	-0.06 (0.089)	0.01 (0.752)	0.06 (0.199)	0.10 (0.026)	-0.24 (0.000)	-0.14 (0.006)
28	Iron	Iron Status	0.02 (0.540)	0.05 (0.148)	0.10 (0.029)	0.08 (0.066)	-0.09 (0.063)	-0.02 (0.725)
29	Iron Binding Capacity	Iron Status	0 (0.996)	-0.01 (0.737)	-0.05 (0.272)	-0.09 (0.041)	0.08 (0.128)	0.10 (0.045)
30	Iron Deficiency	Iron Status	-0.05 (0.210)	-0.10 (0.007)	-0.11 (0.047)	-0.14 (0.013)	0.01 (0.036)	0.06 (0.197)
31	Transferrin	Iron Status	0.01 (0.853)	0 (0.959)	-0.04 (0.422)	-0.07 (0.127)	0.07 (0.147)	0.10 (0.041)
32	Transferrin Saturation	Iron Status	0.01 (0.737)	0.04 (0.269)	0.10 (0.028)	0.10 (0.034)	-0.12 (0.018)	-0.06 (0.261)
33	Calcium	Kidney Function and Electrolytes	-0.03 (0.384)	0.02 (0.485)	-0.03 (0.528)	0.05 (0.259)	-0.04 (0.404)	0.07 (0.179)
34	Creatinine	Kidney Function and Electrolytes	-0.01 (0.671)	-0.08 (0.013)	0 (0.944)	-0.06 (0.158)	-0.05 (0.268)	-0.04 (0.418)
35	Magnesium	Kidney Function and Electrolytes	-0.04 (0.210)	0.02 (0.487)	-0.02 (0.594)	-0.01 (0.857)	-0.11 (0.033)	-0.08 (0.113)
36	Phosphate	Kidney Function and Electrolytes	0 (0.940)	0.02 (0.652)	-0.01 (0.761)	-0.03 (0.468)	0.03 (0.52)	0.05 (0.269)
37	Potassium	Kidney Function and Electrolytes	-0.02 (0.598)	0 (0.895)	-0.04 (0.396)	-0.02 (0.684)	0.02 (0.647)	0.05 (0.268)
38	Sodium	Kidney Function and Electrolytes	0.02 (0.626)	0 (0.958)	0.06 (0.170)	0.04 (0.379)	-0.07 (0.181)	-0.04 (0.478)
39	Urea	Kidney Function and Electrolytes	-0.08 (0.014)	-0.06 (0.096)	-0.08 (0.096)	-0.05 (0.313)	-0.13 (0.008)	0.01 (0.776)
40	Uric Acid	Kidney Function and Electrolytes	-0.07 (0.038)	-0.06 (0.063)	-0.07 (0.135)	-0.08 (0.079)	-0.09 (0.070)	0.02 (0.625)
41	eGFR (Creatinine)	Kidney Function and Electrolytes	0.14 (0.000)	0.09 (0.008)	0.11 (0.008)	0.07 (0.085)	0.23 (0.000)	0.05 (0.339)
42	Cholesterol	Lipids and Cardiovascular health	-0.04 (0.218)	0.04 (0.209)	-0.01 (0.748)	0.04 (0.369)	-0.08 (0.105)	-0.02 (0.745)
43	High-Density Lipoprotein	Lipids and Cardiovascular health	0.03 (0.378)	0.06 (0.088)	0.02 (0.721)	0.03 (0.567)	0.05 (0.317)	0.08 (0.124)
44	Low-Density Lipoprotein	Lipids and Cardiovascular health	-0.04 (0.270)	0.04 (0.216)	0 (0.937)	0.04 (0.333)	-0.08 (0.091)	-0.03 (0.518)
45	Triglycerides	Lipids and Cardiovascular health	-0.06 (0.075)	-0.05 (0.123)	-0.05 (0.296)	-0.02 (0.675)	-0.09 (0.075)	-0.05 (0.357)
46	Alanine Aminotransferase	Liver Biochemistry	-0.01 (0.838)	0.02 (0.482)	0.03 (0.488)	0.05 (0.244)	-0.06 (0.244)	-0.03 (0.559)
47	Albumin	Liver Biochemistry	0.07 (0.039)	0.02 (0.609)	0.09 (0.034)	0.02 (0.673)	0.03 (0.601)	0 (0.983)
48	Alkaline Phosphatase	Liver Biochemistry	-0.03 (0.404)	-0.01 (0.778)	0 (0.925)	0 (1)	-0.09 (0.064)	-0.02 (0.749)
49	Aspartate Aminotransferase	Liver Biochemistry	-0.03 (0.386)	0.01 (0.711)	0.01 (0.771)	0.05 (0.279)	-0.09 (0.063)	-0.05 (0.340)
50	Gamma-Glutamyl Transferase	Liver Biochemistry	-0.07 (0.033)	0.01 (0.874)	-0.06 (0.195)	0.03 (0.529)	-0.10 (0.047)	0.02 (0.762)
51	Lactate Dehydrogenase	Liver Biochemistry	-0.12 (0.001)	-0.05 (0.111)	-0.13 (0.004)	-0.01 (0.763)	-0.12 (0.017)	-0.04 (0.385)
52	Protein total	Liver Biochemistry	-0.04 (0.240)	-0.04 (0.266)	0.02 (0.728)	-0.04 (0.369)	-0.12 (0.014)	-0.08 (0.108)
53	Creatine Kinase	Muscle Damage	-0.01 (0.658)	-0.02 (0.492)	-0.01 (0.771)	-0.05 (0.307)	-0.02 (0.736)	-0.03 (0.560)

Time since blood test

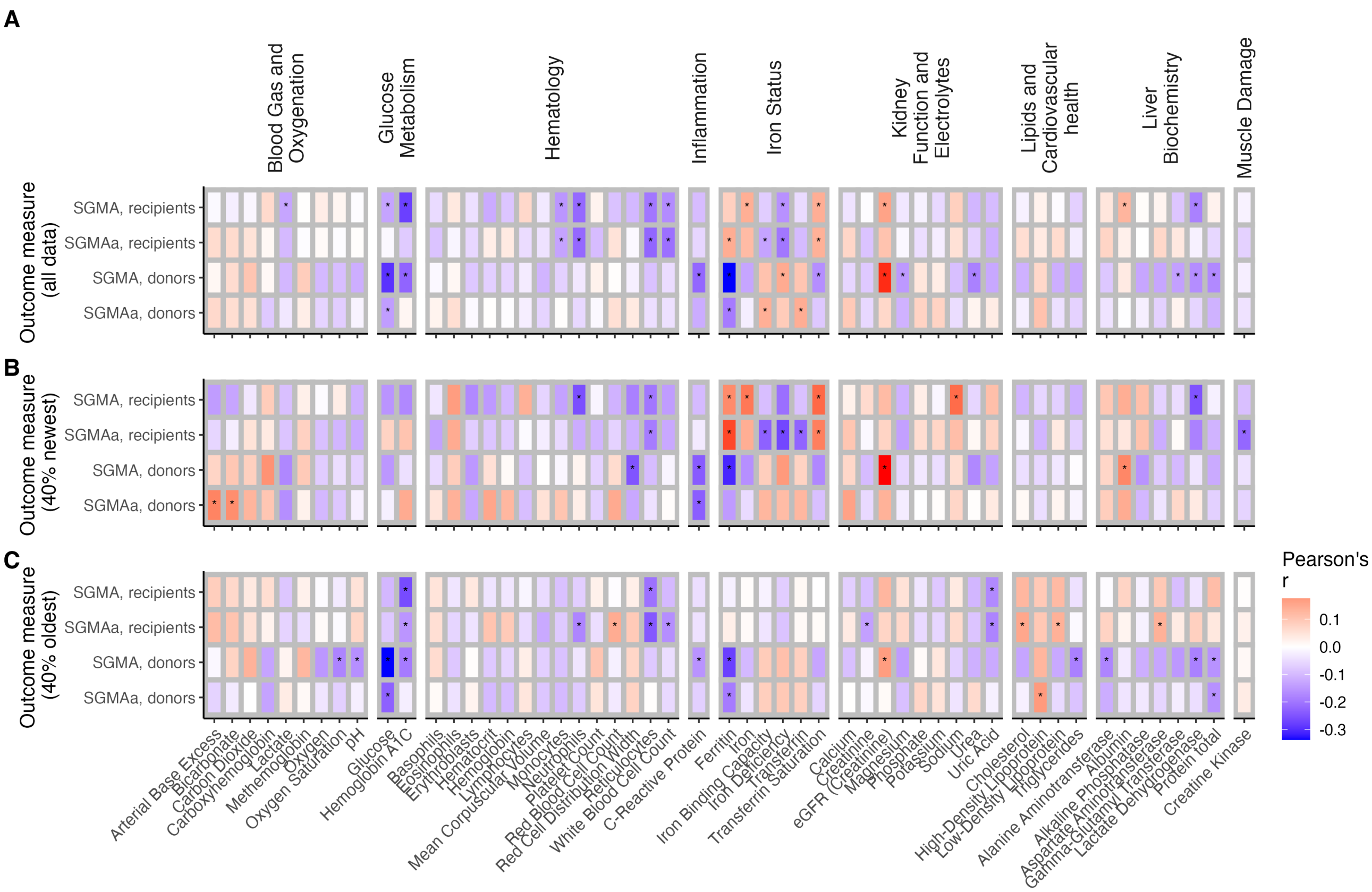

Supplementary Figure 8. **Panel A** shows individual Pearson's correlation coefficients between SGMA scores for recipients, SGMAa scores for recipients, SGMA scores for donors, and SGMAa scores for donors and blood measurements across the full dataset (corresponding to main Figure 5). **Panel B** shows correlations restricted to participants with the most recent blood marker data (40% most recent observations). **Panel C** shows correlations restricted to participants with the oldest assessments (40% oldest observations). Stars indicate significance at the level of $* : p < 0.05$. Sample sizes are given in Supplementary Table 4.
