# Supplementary material for: Computational assessment of memory function in kidney transplant recipients and donors
Source: Commun Med (Lond). 2026 Jun 5;6:480. doi: 10.1038/s43856-026-01663-x (PMC13562709; doi:10.1038/s43856-026-01663-x)
Supplement: Supplementary file 2 [file 43856_2026_1663_MOESM2_ESM.docx]

Description of Additional Supplementary Files

File name: Supplementary Data 1-6

Description: Source data to generate the main figures
